# Formation environments and mechanisms of multistage paleokarst of Ordovician carbonates in Southern North China Basin

**DOI:** 10.1038/s41598-020-80878-x

**Published:** 2021-01-12

**Authors:** Haitao Zhang, Guangquan Xu, Mancai Liu, Minhua Wang

**Affiliations:** 1 School of Earth and Environment, Anhui University of Science and Technology, Huainan, 232001 China; 2 Department of Geology and Hydrogeology, Huaihe Energy Holding Group Co., Ltd., Huainan, 232001 China

**Keywords:** Biogeochemistry, Environmental sciences, Hydrology, Solid Earth sciences, Engineering

## Abstract

With the reduction of oil and gas reserves and the increase of mining difficulty in Northern China, the carbonate rocks in Southern North China Basin are becoming a significant exploration target for carbonate reservoirs. However, the development characteristics, formation stages, formation environments and mechanisms of the carbonate reservoirs in Southern North China Basin are still unclear, which caused the failures of many oil and gas exploration wells. This study focused on addressing this unsolved issue from the Ordovician carbonate paleokarst in the Huai-Fu Basin, which is located in the southeast of Southern North China Basin and one of the key areas for oil and gas exploration. Based on petrology, mineralogy and geochemical data, pore types, distribution characteristics, and formation stages of the Ordovician paleokarst were analyzed. Then, in attempt to define the origins of porosity development, the formation environments and mechanisms were illustrated. The results of this study showed that pore types of the Ordovician carbonates in the Huai-Fu Basin are mainly composed of intragranular pores, intercrystalline (intergranular) pores, dissolution pores (vugs), fractures, channels, and caves, which are usually in fault and fold zones and paleoweathering crust. Furthermore, five stages and five formation environments of the Ordovician paleokarst were identified. Syngenetic karst, eogenetic karst, and paleoweathering crust karst were all developed in a relatively open near-surface environment, and their formations are mainly related to meteoric water dissolution. Mesogenetic karst was developed in a closed buried environment, and its formation is mainly related to the diagenesis of organic matters and thermochemical sulfate reduction in the Permian-Carboniferous strata. Hydrothermal (water) karst was developed in a deep-buried and high-temperature environment, where hydrothermal fluids (waters) migrated upward through structures such as faults and fractures to dissolve carbonate rocks and simultaneously deposited hydrothermal minerals and calcites. Lastly, a paleokarst evolution model, combined with the related porosity evolution processes, nicely revealed the Ordovician carbonate reservoir development. This study provides insights and guidance for further oil and gas exploration in the Southern North China Basin, and also advances our understanding of the genesis of carbonate paleokarst around the world.

## Introduction

Carbonate karst is of great importance for carbonate karst reservoirs with high oil and gas reserves, including 52% of the reserves of oil and gas and about 60% of oil and gas outputs in the world^1^. Among the eight major oil and gas basins in the world, except for the Sichuan Basin in China and the East Siberian Basin in Russia, the oil and gas reservoirs in the Tarim Basin, Ordos Basin, Permian Basin, Williston Basin, Michigan Basin, and the Oman Basin, are all related to the Lower Paleozoic Ordovician and/or Cambrian carbonate paleokarst^1–3^. Therefore, an in-depth study of the developmental characteristics and formation mechanism of the Lower Paleozoic carbonate paleokarst will provide great insights and guidance for scientific exploration, development, and management of major oil and gas basins in the world.

In Northern China, oil and gas reserves are mainly concentrated in the Lower Paleozoic Ordovician carbonates in the Tarim Basin, Ordos Basin, and Bohai Bay Basin, where more than 60% of the total oil and gas production in China comes from^1,3^. Many researchers have found that the Ordovician carbonates in Northern China recorded not merely one, but multistage paleokarst, and different regions have rather site-specific formation environments and stages of the Ordovician paleokarst^4–6^. For example, Zhang et al.^7,8^ identified four different formation environments of the Ordovician paleokarst in the Tarim Basin based on the analysis of carbon and oxygen isotopes and fluid inclusion, that is, a marine environment during syndiagenesis karst stage, an atmospheric freshwater environment during paleoweathering crust karst stage, a shallow-burial environment during mesogenetic karst stage, and a deep-burial and high-temperature environment during hydrothermal karst stage. Qing et al.^9^ determined that there are three stages of paleokarst in the Ordovician carbonates in the Ordos Basin through oxygen and carbon isotopes and fluid inclusion microthermometry, that is, paleoweathering crust karst formed by meteoric water dissolution, hydrothermal karst formed by fluid-rock reaction, and mesogenetic karst formed by formation hydrocarbon generation and pressure-released water. Moreover, some researchers^10–12^ employed thin sections, carbon and oxygen isotopes, fluid inclusion, and other methods to find four stages of paleokarst in the Ordovician carbonates in the Bohai Bay Basin, namely paleoweathering crust karst formed by meteoric water dissolution, mesogenetic karst formed by formation hydrocarbon generation, network fracture karst formed by multistage tectonic activities, and hydrothermal karst formed by hydrothermal activities.

The Southern North China Basin belongs to the southern part of the North China Platform, where the southern part is bordered by the Qinling Dabie orogenic belt, the northern part is bordered by the Jiaozuo-Shangqiu faults, the eastern part is bordered by Tanlu fault, and the western part is bordered by the Yichuan-Qishan-Gushi-Feizhong faults^13^. Since the Late Paleozoic, the sedimentary environments, diagenetic processes, tectonic evolution, and hydrogeological conditions in the Southern North China Basin are quite different from those of the Tarim Basin, Ordos Basin, and Bohai Bay Basin because the study area is close to the edge of the north–south plate in China^13–15^, the formation and evolution of the Ordovician carbonate paleokarst in the Southern China North Basin are also different from those basins. However, the development characteristics, formation environments and mechanisms of the Ordovician carbonate paleokarst in the Southern North China Basin have never been studied in details before, which might be one of the reasons for the failures of many oil and gas exploration wells in this Basin in the past.

The Huai-Fu Basin, including Huainan and Fuyang regions, is located in the southeast margin of the Northern China plate^16^. This is one of the important exploration regions for the Ordovician carbonate reservoirs in the Southern North China Basin. Like other areas in Southern North China Basin, the Ordovician carbonates in the Huai-Fu Basin are widely developed, but the burial depth is relatively shallow because it is near the edge of the Northern China Plate^16^, thus the costs for exploration and study are lower. Moreover, there are two complete outcrops (Shungeng Mountain and Bagong Mountain) of the Ordovician carbonates in the east of the study area, which greatly facilitates field investigations and observations. Therefore, we chose the Ordovician carbonates in the Huai-Fu Basin as the field site in this study.

Using petrology, mineralogy, and geochemical data from outcrops, drilling cores, thin sections, carbon and oxygen isotopes, and minor elements, this study aims to achieve the following objectives: (1) showing the paleokarst characteristics of the Ordovician carbonate rocks in the Huai-Fu Basin, (2) identifying and evaluating the formation stages and environments of the Ordovician paleokarst based on paleokarst morphology and geochemical characteristics, (3) revealing the formation mechanisms of multistage paleokarst, and (4) establishing the evolution models of the Ordovician carbonate paleokarst in the study area.

## Geologic setting

**Figure 1:**
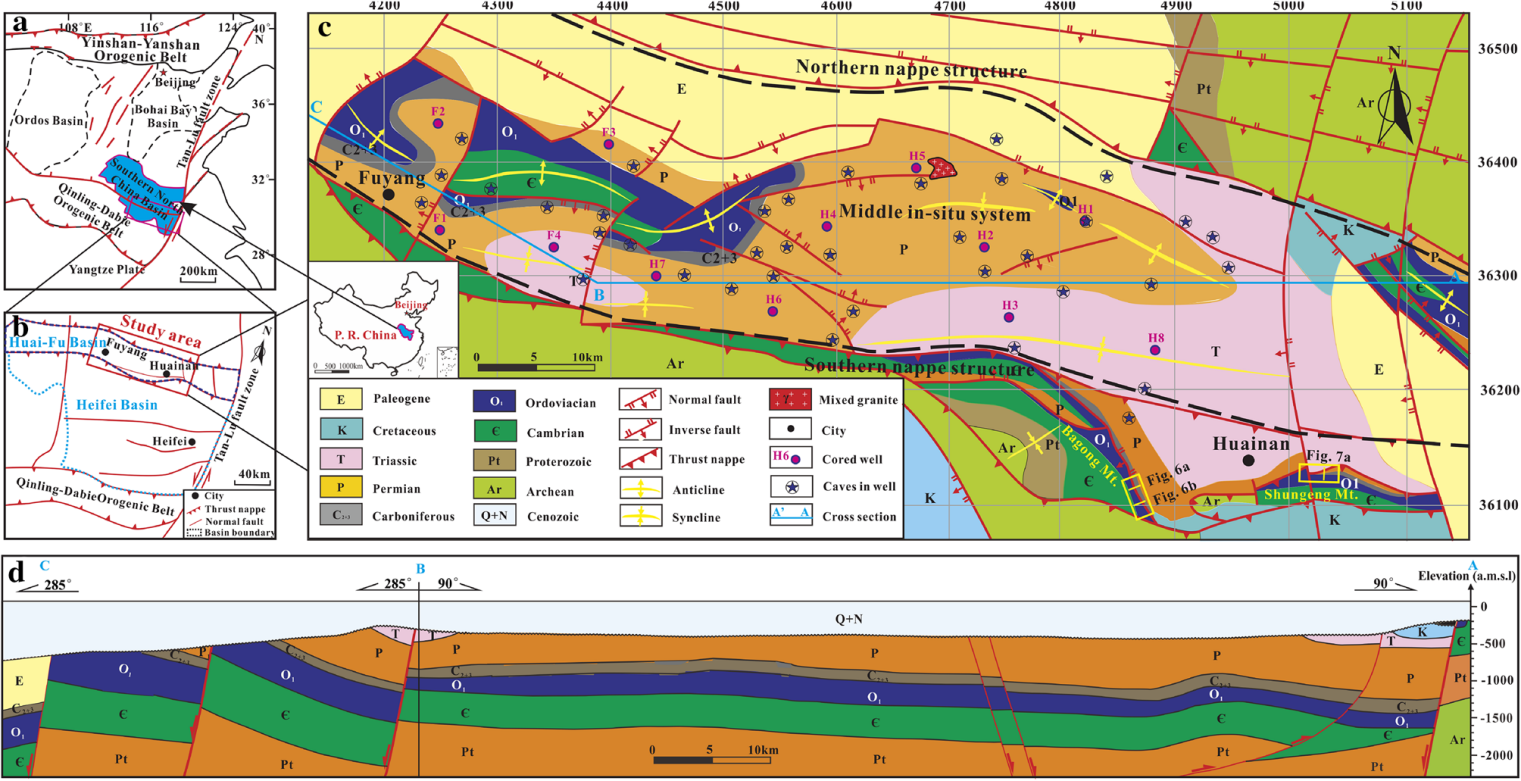
Geological setting of the studied area: (**a**) Distribution of major oil and gas basins in North China; (**b**) Location of the study area; (**c**) Strata, structures and well locations in the Huai-Fu Basin; (**d**) Geological map of cross section A-B-C in the Huai-Fu Basin of China. Maps created by the authors with ArcGIS Pro 2.6.1 (https://www.esri.com/de-de/arcgis/products/arcgis-pro/overview). Note: (**c**) is a geological map of the bedrock in the study area, so the Cenozoic loose layer overlying the bedrock cannot be displayed; (**d**) is a geological section, so the Cenozoic loose layer overlying the bedrock can be displayed.

The Huai-Fu Basin is located in the southeast of the Southern North China Basin (Fig. 1a) and the north of the Hefei Basin (Fig. 1b), which measures an area of 3,500 km^2^. The Huai-Fu Basin can be divided into three tectonic units, in which the north and south are thrust structures, and the middle is in-situ system (Fig. 1c). Many rooted faults and folds (Fig. 1c,d) in this area were developed due to multistage tectonic activities during the Indochina, Yanshan, and Himalayan tectonic stages^16,17^. The Indochina tectonic stage caused north–south compression in the study area, forming a nappe structure; the Yanshan and Himalayan tectonic stages not only reactivated the pre existing faults but also produced many NNE–SSW and NNW–SSE striking normal faults, accompanied by multiple phases of magmatic and volcanic activities^16,17^.

The Huai-Fu Basin mainly experienced three periods of magmatic and volcanic activities during the Late Triassic (240 Ma), Middle Jurassic (140 Ma), and Early Cretaceous (120 Ma)^18^. In the central part of the study area, magmatic intrusive rocks with an area about 16 km^2^ have been found in the Permian strata (Fig. 1c)^19^. Xu et al.^20^ showed that this magmatic activity might take place in the Early Cretaceous, and its intrusion time was about 115–130 Ma based on U–Pb isotopic dating of zircon grains from diabase.

The Huai-Fu Basin contains Paleozoic shallow marine and coastal sediments and Mesozoic and Cenozoic terrestrial sediments, overlying the Precambrian metamorphic basement^16^. Like other areas in Northern China, the Lower Paleozoic Ordovician carbonates in the study area are also the focus of oil and gas exploration.

**Figure 2:**
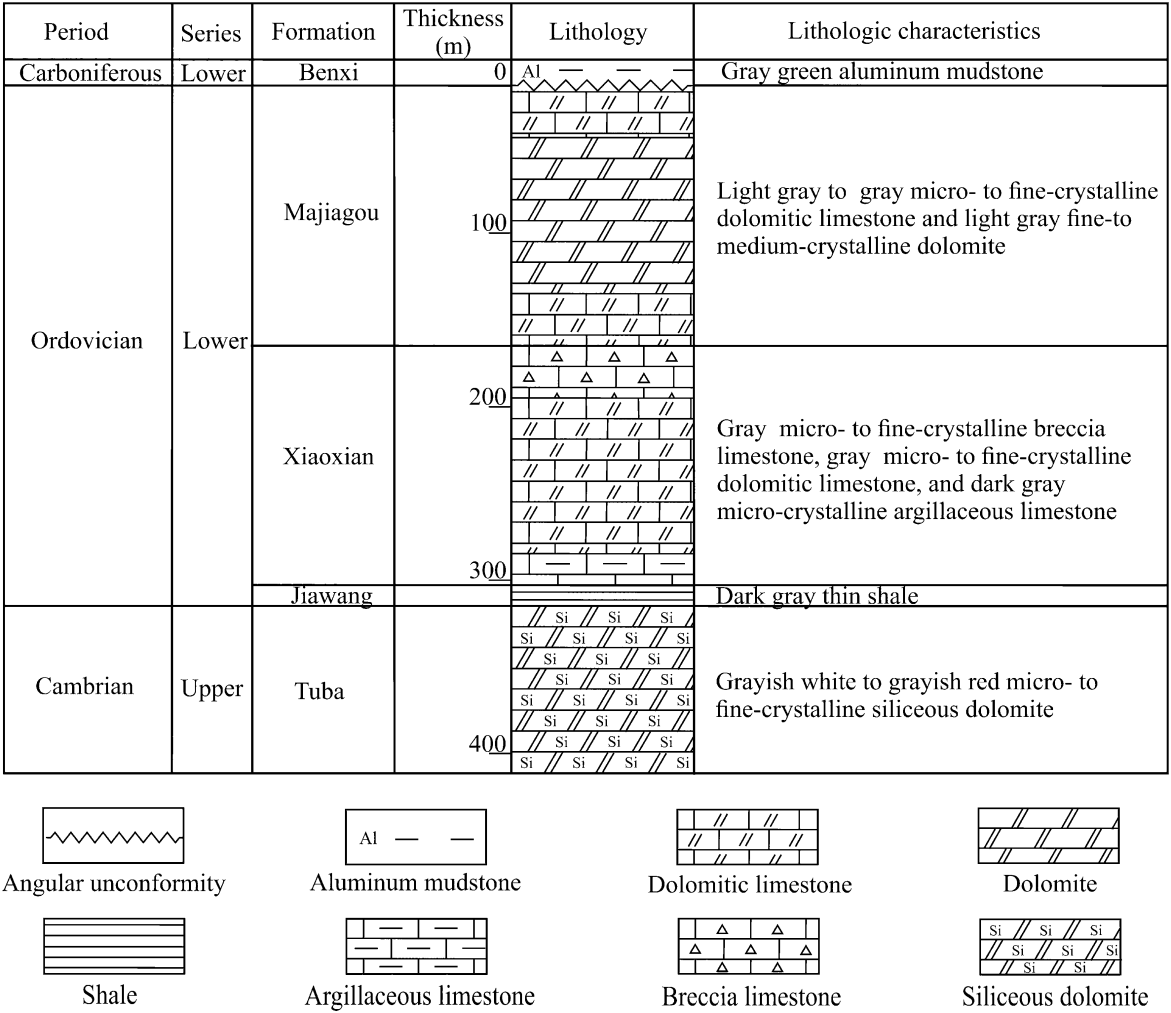
The Ordovician stratigraphic column of the in-situ system in the Huai-Fu Basin.

The Ordovician sediments are mainly composed of limestones and dolomites (Fig. 2), which are widely distributed in the study area (Fig. 1c,d). The Shungeng Mountain and Bagong Mountain outcrops (Fig. 1) in the east part of the study area expose the entire Ordovician stratigraphic section. The thickness of the Ordovician carbonates in the outcrops changed greatly due to the influence of faults and folds, ranging from 69 to 600 m thick. The exploration wells in the middle part of the Huai-Fu Basin show that the Ordovician strata in the middle in-situ system are about 300 m thick. From top to bottom, the Ordovician strata are composed of three formations: Lower Ordovician Majiagou Formation, Lower Ordovician Xiaoxian Formation, and Lower Ordovician Jiawang Formation, as shown in Fig. 2. The Upper and Middle Ordovician Formations were not developed in the study area or were eroded by weathering and dissolution during the Ordovician paleoweathering period^21^. The Majiagou Formation is mainly composed of thick dolomitic limestones and dolomites (Fig. 2), with an average thickness of 150 m; the Xiaoxian Formation is primarily composed of thin to medium-thick breccia limestones, dolomitic limestones, and argillaceous limestones, with an average thickness of 138 m (Fig. 2); the Jiawang Formation is composed of thin shales (Fig. 2), with an average thickness of 12 m. These sediments were mainly developed in the tidal flats and subtidal environments.

The Cambrian strata are under the Ordovician sediments, with approximately 1060 m thick according to the Cambrian outcrops in the eastern part of the study area (Fig. 1c). The Upper Cambrian Tuba Formation is primarily composed of siliceous dolomites, and the Middle and Upper Cambrian mainly consists of dolomites, dolomitic limestones, and shales, where the paleokarst is generally not developed. The Carboniferous Benxi Formation covers over the Ordovician paleoweathering crust, which is mainly composed of aluminum mudstones, with an average thickness of 20 m. Upward are the Carboniferous Taiyuan Formation and Permian strata, which are composed of coal seams, thin-layer limestones, mudstones, and sandstones. The Mesozoic and Cenozoic deposits mainly consist of terrestrial sandstones and mudstones.

## Samples and methods

Approximately 60 exploration wells completely penetrated the Ordovician carbonate strata in the Huai-Fu Basin. These wells are distributed across the entire study area and mainly in the form of cores and drilling data provide a solid constrain for the presented study. In this study, the observation and test data from the Ordovician outcrop (Shungeng Mountain and Bagong Mountain, see Fig. 1c) and 12 representative wells (Fig. 1c) with a total core length of 1243.56 m were selected for detailed analysis. These core samples are primarily composed of carbonate matrix (CM) and fillings (Fs), where fillings include pore and vug fillings, fracture fillings, channel fillings, and cave fillings.

One hundred and thirty-two samples for the thin section observation were polished to about 0.04 mm thick. Microscopic observations of thin sections for petrology, mineralogy and pore structure were analyzed in the State Key Laboratory of Anhui University of Science and Technology using an Olympus CX41 microscope (Shinjuku, Tokyo, Japan).

Forty-eight samples for the carbon and oxygen isotope analyses were carried out on a Kiel IV carbonate device (Thermo Fisher Scientific, Bremen, Germany) connected to a Mat 253 mass spectrometer (Thermo Finnigan, Bremen, Germany) in the Geochemistry and Isotope Laboratory of Southwest University. Approximately 200 mg of each powdered sample was placed in a reaction bottle which was connected to a vacuum system. Each sample was reacted with 100% H_3_PO_4_ at 70 °C to generate CO_2_, which was collected in Trap1 with liquid nitrogen at – 196 °C. Then, Trap1 was heated to – 90 °C, and the released CO_2_ was transferred to Trap2. The Trap2 was then heated to 30 °C, and the released CO_2_ was carried into a Mat 253 mass spectrometer for analysis. All the carbon and oxygen isotope values were reported relative to Pee Dee Belemnite (PDB) and calibrated against NBS-18 and NBS-19 standards. Reproducibility of replicate analyses of the carbon and oxygen isotopes for NBS-18 and NBS-19 standards were ± 0.1‰ and ± 0.2‰, respectively.

The forty-eight samples for the carbon and oxygen isotopes testing were also used for the minor elements testing. The testing was performed via ICP-MS (Agilent 4500, Agilent Ltd, Cheshire UK) using an ICP-OES (Spectro Analytical CIROS, Kleeve, Germany) in the Geochemistry and Isotope Laboratory of Southwest University. Approximately 300 mg of each powdered sample was placed in a jar containing 3 ml (1 + 1) HNO_3_. The jar was put on a hot plate, and the temperature was maintained at 120 °C for 24 h, and then the temperature was increased to 150 °C and maintained at this temperature for another 24 h. After the solution was evaporated to almost dryness, 2 ml (1 + 1) HNO_3_ was added, and the plate was kept on a hot plate for 2 h. The solution was evaporated to 1 ml, then transferred to a 50 ml polyethylene bottle. Prior to analysis, the solution was diluted to 20 g with sub-boiling water.

## Results

### Petrology and mineralogy

**Figure 3:**
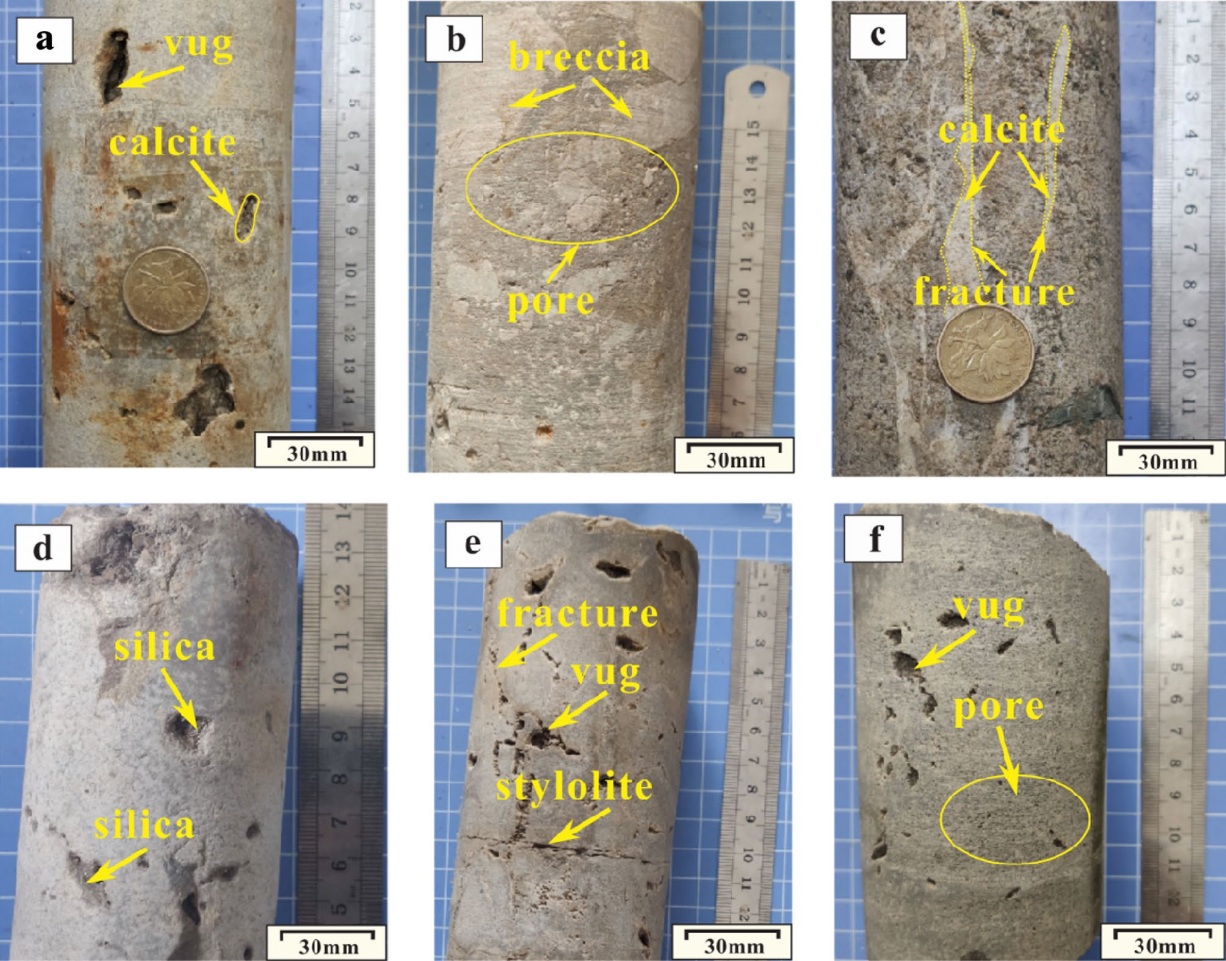
Photos of the Ordovician carbonate rocks and paleokarst: (**a**) Dolomitic limestones with vugs semi-filled with calcites, Well H1, 522.14 m; (**b**) Breccia limestones with pores, Well H6, 1367.50 m; (**c**) Argillaceous limestones with fractures fill-filled with calcites, Well H3, 1555.70 m; (**d**) Dolomites with vugs semi-filled with silica, Well F4, 1425.64 m; (**e**) Calcite dolomites with vugs, fractures and stylolites, Well F1, 672.05 m; (**f**) Argillaceous dolomites with vugs and pores, Well F3, 1236.50 m.

**Figure 4:**
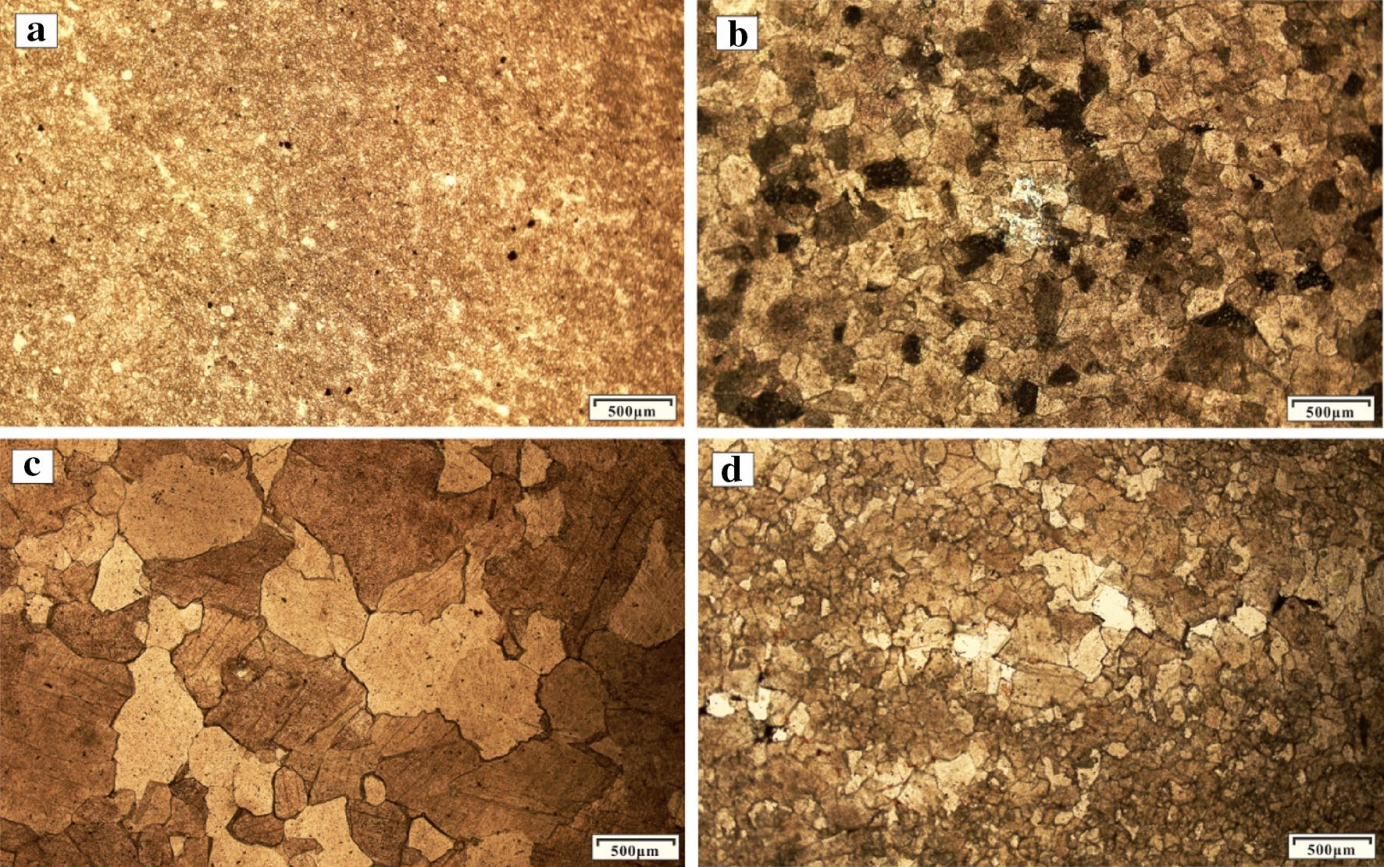
Microphotographs of the Ordovician carbonate crystals (particles): (**a**) Micro-crystalline argillaceous limestones, Well H2, 1089.35 m; (**b**) Medium crystalline dolomites, Well F4, 1456.85 m; (**c**) Coarse crystalline breccia limestones, Well F5, 757.80 m; (**d**) Medium-fine crystalline dolomitic limestones, Well F2, 1134.87 m.

The Ordovician section in the Huai-Fu Basin is mainly composed of dolomitic limestones (Fig. 3a), breccia limestones (Fig. 3b), argillaceous limestones (Fig. 3c), and dolomites (Fig. 3d), with a small amount of calcite dolomites (Fig. 3e) and argillaceous dolomites (Fig. 3f). Based on observations of thin sections, the Ordovician carbonates are mainly composed of micro to fine-medium to medium-coarse crystalline limestones and dolomites, as shown in Fig. 4a–d. From micro to fine-medium to medium-coarse crystalline, the color of carbonate rocks gradually changes from dark gray (Fig. 3f) to gray (Fig. 3e), and then to light gray (Fig. 3d). The crystal shapes in the micro and fine crystalline carbonate rocks cannot be easily identified because the crystal sizes are less than 100 μm (Fig. 4a,b,d). The sizes of the medium to coarse crystals are generally between 100 and 2000 μm (Fig. 4c), and most of them contain euhedral and subhedral crystals and always display poikilitic textures, which make it easy to identify them.

The dolomitic limestones are dominated by micro to medium-fine crystal calcites (Figs. 3a, 4d), containing a large number of micro and fine crystalline dolomite, and the calcite contents exceed 60%. The breccia limestones are mainly composed of limestone breccias, which are filled and cemented by calcites (Fig. 3b). The size of the breccias ranges from 1 to 100 mm, with poor sorting, good roundness, and sub roundness, showing that these breccias were cemented after the long-term dissolution of groundwater^22^. The calcite contents in the breccia limestones vary widely, ranging from 50 to 80%. The argillaceous limestones are composed of micro calcite less than 50 μm (Figs. 3c, 4a), which contains a small amount of dolomites and biological debris, and the calcite contents are more than 80%. The dolomites are dominated by fine to medium-fine crystal dolomites (Figs. 3d, 4b), and the dolomite contents are more than 80%.

### Pore types and characteristics

The pore types in the Ordovician carbonate rocks in the Huai-Fu Basin are principally intragranular pores, intercrystalline (intergranular) pores, dissolution pores (vugs), fractures, channels, and caves.

**Figure 5:**
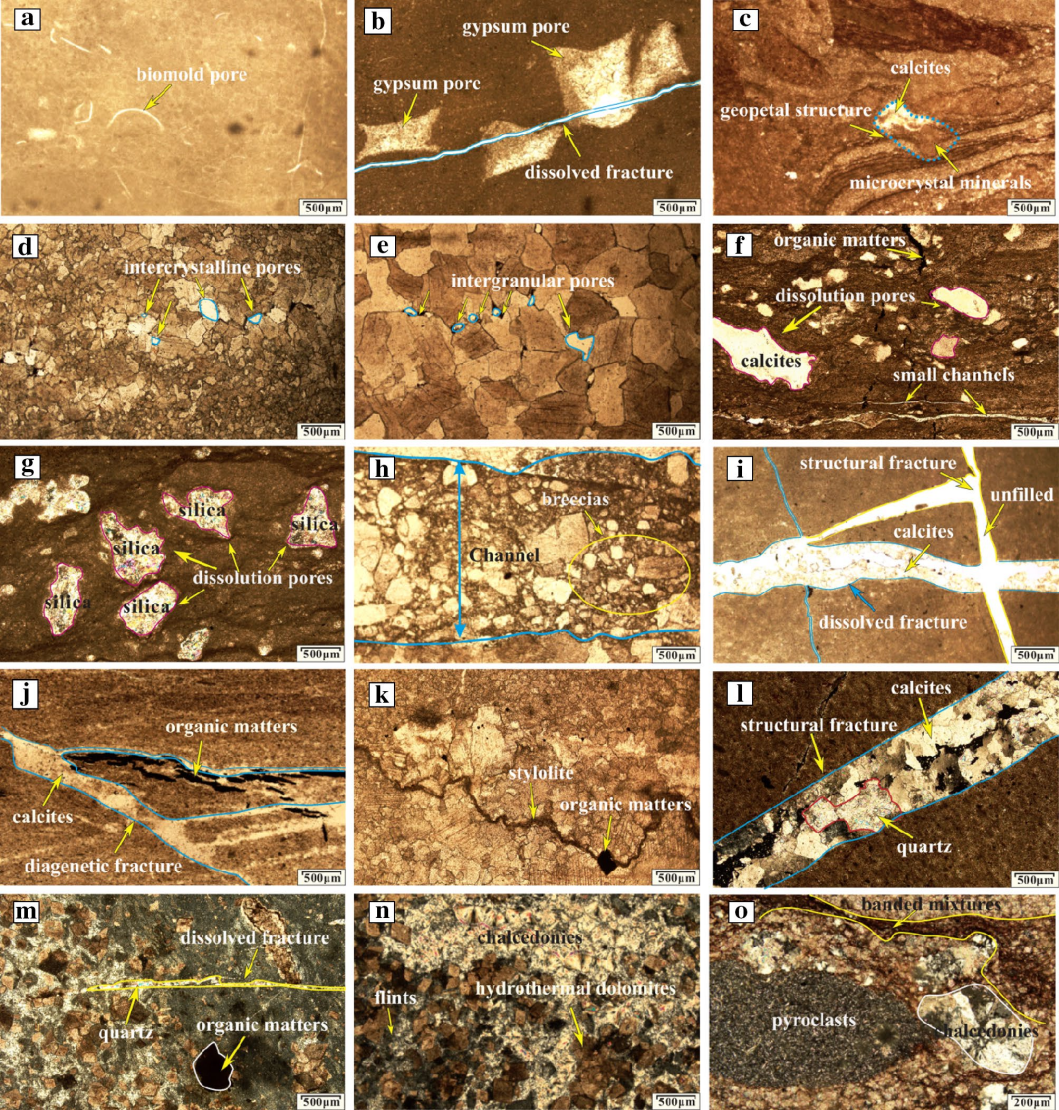
Microphotographs of the Ordovician carbonate pores and fillings: (**a**) Biomold pores full-filled with calcites, Well H8, 1654.07 m; (**b**) Gypsum pores and dissolved fractures full-filled with calcites, Well H3, 1545.15 m; (**c**) Geopetal structure: the upper part is filled with bright calcites and the lower part is filled with dark materials, Well H6, 1478.35 m; (**d**) Intercrystalline pores full-filled with calcites, Well F1, 870.05 m; (**e**) Intergranular pores full-filled with calcites, Well F2, 1103.57 m; (**f**) Dissolution pores, fractures and small channels filled with calcites or organic matters, Well H1, 508.13 m; (**g**) Dissolution pores filled with silica or organic matters, Well H4, 1070.85 m; (**h**) Channels filled with karst breccias, Well H1, 660.45 m; (**i**) Tectonic fractures and dissolved fractures, Well F3, 1438.95 m; (**j**) dissolved fractures full-filled with calcites and organic matters, Well H1, 580.25 m; (**k**) Stylolites full-filled with organic matters, Well H2, 1120.67 m; (**l**) dissolved fractures semi-filled with quartzs and calcites, Well F1, 863.62 m; (**m**) Dissolution pores and fractures filled with thermal minerals, Well H5, 855.34 m; (**n**) Thermal minerals, Well H5, 913.67 m; (**o**) Thermal minerals, Well H5, 937.35 m.

The intragranular pores, including biofilm pores (Fig. 5a) and gypsum pores (Fig. 5b), lie in microcrystal limestones and are cemented with calcites. In addition, there is a special filling structure for the intragranular pore, called the geopetal structure (Fig. 5c)^23^. The geopetal structure includes a lower part of the dissolved grain cemented with dark minerals (Fig. 5c), and an upper part cemented with light calcites (Fig. 5c).

The intercrystalline or intergranular pores refer to the pores between mineral crystals in carbonate rocks, in which intergranular pores (Fig. 5d) refer to the pores between dolomite grains in dolomites while intergranular pores (Fig. 5e) refer to the pores between calcite grains in limestones. They generally lie in the fine and medium crystalline limestones or dolomites and are completely filled with calcites. The sizes of such pores range from tens to hundreds of millimeters.

The dissolution pores and vugs are usually developed in the dolomitic limestones and dolomites. Macroscopically, the dissolution vugs are alveolar-shaped pores with the size between 50 to 300 mm, and most are semi-filled with calcites (Fig. 3a,e,f) or full-filled with silica (Fig. 3d); microscopically, the dissolution pores are irregular pores with a diameter of 1–2 mm and are full-filled with calcites (Fig. 5f), and they may also include organic matters (Fig. 5f,g) and silica (Fig. 5g). The Ordovician carbonate rocks with dense dissolution vugs usually show honeycomb textures (Fig. 3a,e,f).

The fractures can be divided into structural fractures (Fig. 5i,l), dissolved fractures (Fig. 5b,i,m), and diagenetic fractures (Fig. 5j,k). (1) Structural fractures. The multi-stage tectonic activities formed two types of structural fractures: one is high angle tensile fractures formed by region stress release, and the other is associated with fault activity and mainly distributed on both sides of the fault zones and in the axial portion of the folds (Fig. 1c,d). According to the stratigraphic occurrence (near E-W striking and dip angle 0° –20°) and fracture characteristics, the structural fractures in the study area can be subdivided into bedding fractures (dip angle 0° –20°), low-angle cracks (dip angle 20°–40°), high angle fractures (dip angle 40° –70°), and vertical fractures (dip angle 70°–90°). The width of bedding and low-angle fractures are less than 0.1 mm, and most of them are full-filled with calcites and/or quartz (Fig. 5i,m). The width of high-angle and vertical fractures are 0.1 mm–5 cm, unfilled (Fig. 5i) or semi-filled with calcites, muds, and/or hydrothermal minerals (Fig. 5l). (2) Dissolved fractures. The dissolved fractures were formed by infiltration and dissolution of aggressive fluids (atmospheric water or hydrothermal fluids) along with the structural fracture system, irregular and uneven fracture surface. Meteoric water and hydrothermal fluids directly infiltrate along micro-cracks, infiltration of calcites, muds, hydrothermal minerals, and so on, which can be seen in the fractures (Fig. 5b,i,m). (3) Diagenetic fractures. Diagenetic fractures are cracks formed from sedimentation to consolidation diagenesis. The shrinkage crack, whose width is generally at the micron scale, formed at the early diagenetic stage with irregularly networked and often filled with calcites (Fig. 5j). Stylolites are the result of the selective dissolution of mineral particles, which is caused by the combined action of overburden pressure and pore fluid under deep burial conditions. Stylolites are mainly formed by horizontally stretching, and usually filled with muds and/or organic matters (Fig. 5k).

**Figure 6:**
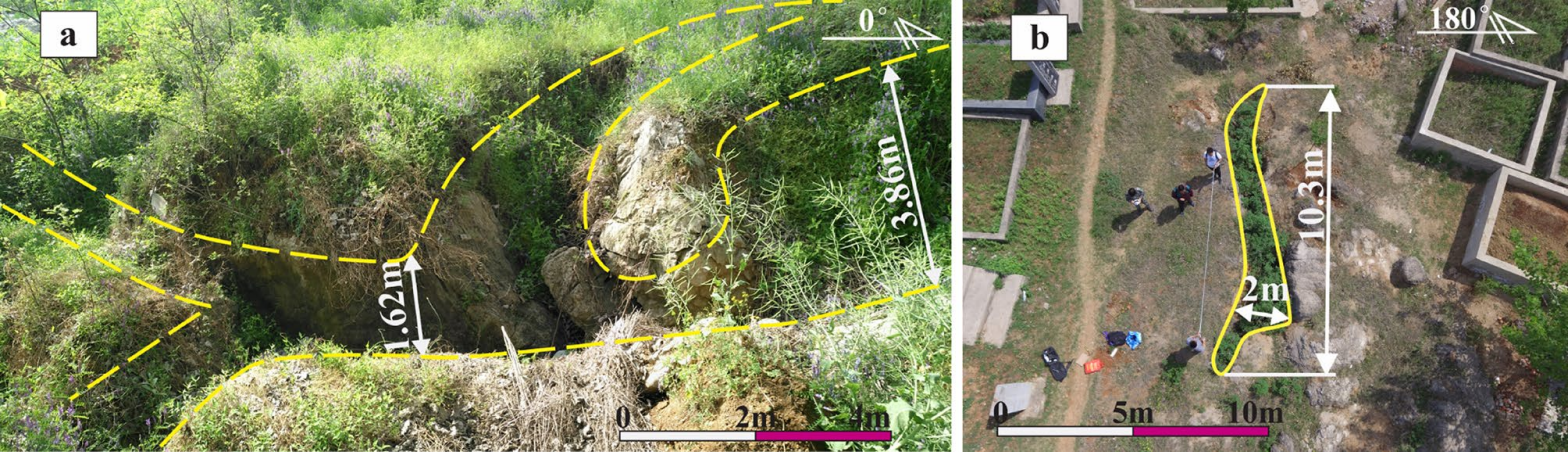
Photos of dissolution channels in outcrop areas: (**a**) Dissolution channels in the Ordovician Majiagou Formation in Bagong Mountain (see Fig. 1c); (**b**) Dissolution channels in the Ordovician Majiagou Formation in Bagong Mountain (see Fig. 1c).

The dissolution channels are solution-enlarged fractures and may be the result of dissolution. They are often observed around tectonic fractures, faults, bedding planes or unconformities, which may be the result of dissolution and expansion of fractures^24^. The opening of the channels ranges from tens of millimeters (Fig. 5f) to several meters (Fig. 6a,b), and the lengths range from several centimeters (Fig. 5f) to several meters (Fig. 6a,b). In underground burial areas, channels are usually semi-filled or full-filled with karst breccias (Fig. 5h) and/or muds and/or calcites (Fig. 5f); while in surface outcrops, channels are usually unfilled or semi-filled with the Quaternary loose sediments, thereby forming surface subsidence ditches (Fig. 6a,b).

**Figure 7:**
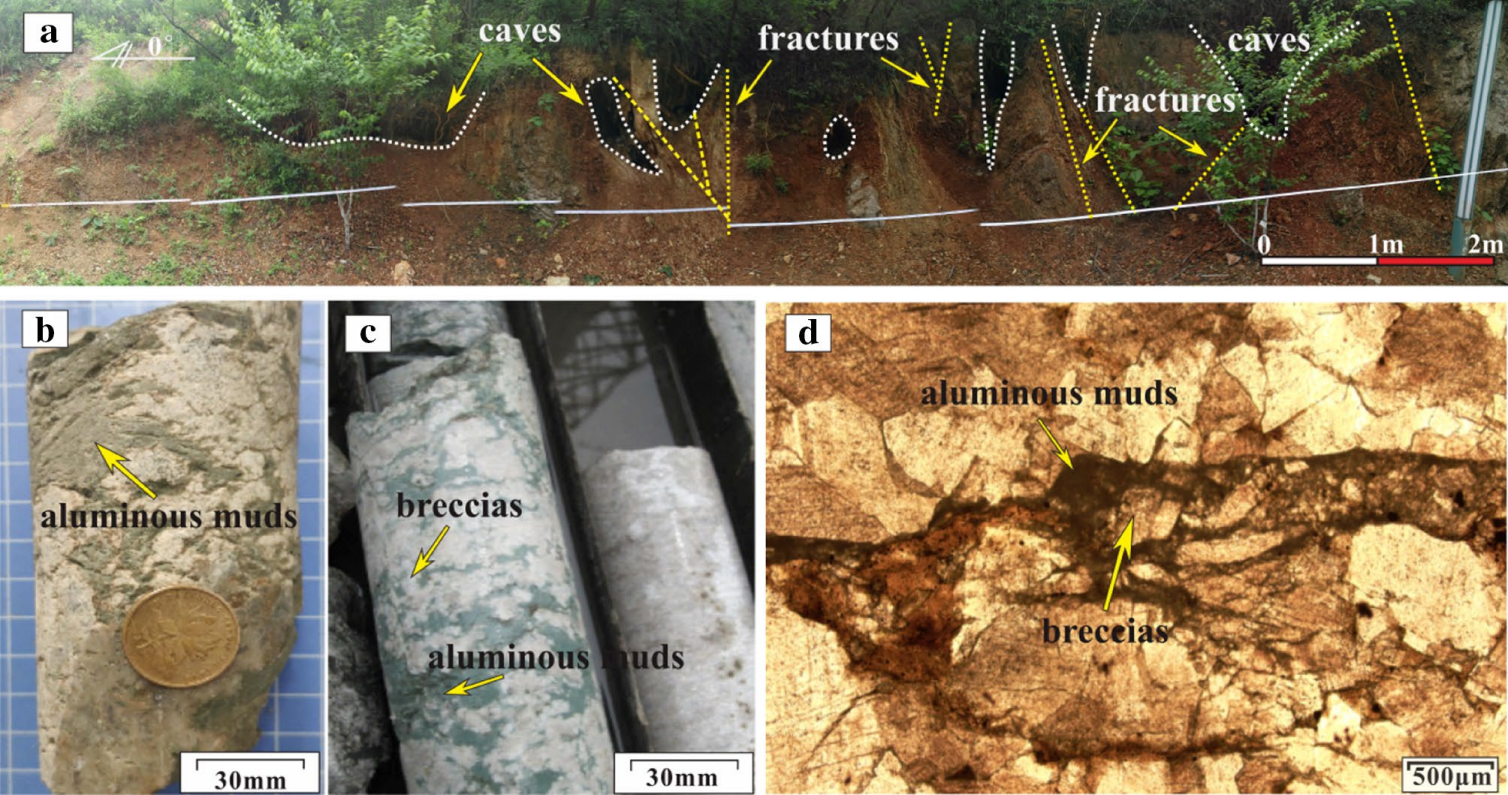
Photos of Ordovician paleoweathering crust karst: (**a**) Caves and fractures in the Ordovician paleoweathering crust in Shungeng Mountain (see Fig. 1a); (**b**) Caves filled with aluminous muds, Well H1, 502.31 m; (**c**) Caves filled with karst breccias and aluminous muds, Well H8, 1423.05 m; (**d**) Thin section of Fig. 7c, Well H8, 1423.05 m.

The caves are primarily developed around the Ordovician paleoweathering crust (Fig. 7a) and faults and fold zones (Fig. 1c), and their sizes vary from a few centimeters to a dozen meters (Fig. 7a). The outcrops and exploration wells show that the cavity fillings and filling patterns are complicated and diversified. Caves in the paleoweathering crust are generally developed within 30 m below the Ordovician unconformities. They are usually filled and cemented by collapse breccias and gray-green muds (Fig. 7b,c,d). Seen from exploration data, caves are usually found near the fault and fold zones (Fig. 1c), and they are generally unfilled or semi-filled with broken surrounding rocks, and sometimes collapse breccias and calcites as well. The obvious blowdown and leakage phenomenon in the drilling process are an important symbol of caves.

### Geochemistry

The carbon and oxygen isotopes and minor elements of 5 carbonate matrix (CM), and 43 fillings (Fs) from the dissolution pores, vugs, fractures, channels, and caves were measured. The results are listed in Table 1.

**Table 1:** Geochemical results for the Ordovician carbonate matrix (CM) and fillings (Fs).

Sample no	Well	Well depth (m)	Materials	Carbon and oxygen isotopes (‰)		Minor elements (ppm)			Formation environment			HCA result
				δ^13^C_PDB_	δ^18^O_PDB_	Ba	Mn	Sr	Paleosalinity	Paleotemperature (°C)	Paleodepth (m)	
1	H1	623.45	Carbonate matrix (CM)	− 1.62	− 5.7	287.65	43.5	90.4	121.14	30.44	211.43	Group A
2	H4	1037.70	Carbonate matrix (CM)	− 2.37	− 5.75	315.73	48.28	68.81	119.58	30.69	219.71	
3	H6	1421.46	Carbonate matrix (CM)	− 2.88	− 6.92	273.54	36.12	88.17	117.96	36.64	418.10	
4	F2	981.63	Carbonate matrix (CM)	− 1.93	− 6.95	261.21	32.82	79.73	119.89	36.80	423.31	
5	F4	1289.67	Carbonate matrix (CM)	− 1.54	− 5.18	283.09	31.67	82.47	121.57	27.89	126.36	
aver				− 2.07	− 6.10	284.24	38.48	81.92	120.03	32.49	279.78	
6	H6	1432.78	Calcites (Fs)	− 1.93	− 5.95	261.91	42.4	83.83	120.38	31.69	252.98	
7	H8	1563.02	Calcites (Fs)	− 2.86	− 6.1	256.73	47.83	93.83	118.40	32.44	278.10	
8	F1	761.30	Calcites (Fs)	− 1.87	− 5.57	303.37	36.53	94.56	120.70	29.80	190.00	
9	H1	511.13	Calcites (Fs)	− 4.63	− 6.53	161.98	23.04	47.96	114.57	34.63	350.96	Group B
10	H1	520.05	Calcites (Fs)	− 5.57	− 6.62	167.66	25.58	36.49	112.60	35.09	366.36	
11	H2	906.23	Calcites (Fs)	− 4.54	− 7.66	145.24	19.12	46.77	114.19	40.55	548.29	
12	H2	1010.13	Calcites (Fs)	− 6.54	− 8.38	138.6	17.37	42.29	109.73	44.45	678.47	
13	H3	1302.65	Calcites (Fs)	− 5.72	− 5.72	150.29	16.76	43.74	112.74	30.54	214.74	
14	H3	1321.02	Calcites (Fs)	− 6.81	− 8.45	139.08	22.46	44.46	109.15	44.84	691.31	
15	H4	913.56	Calcites (Fs)	− 7.7	− 5.77	136.32	25.34	49.78	108.66	30.79	223.02	
16	H5	707.65	Calcites (Fs)	− 4.72	− 6.78	107.95	19.34	50.16	114.26	35.92	393.88	
17	H5	713.63	Calcites (Fs)	− 5.82	− 7.89	147.64	21	43.71	111.45	41.79	589.50	
18	H6	1289.23	Calcites (Fs)	− 4.62	− 6.99	152.82	23.32	33.26	114.36	37.01	430.26	
19	H7	1203.45	Calcites (Fs)	− 6.87	− 8.07	132.39	17.43	42.63	109.21	42.76	622.00	
20	H8	1436.17	Calcites (Fs)	− 7.3	− 7.15	126.33	15.83	38.54	108.79	37.85	458.18	
21	F1	652.18	Calcites (Fs)	− 6.61	− 5.95	136.99	15.27	39.87	110.80	31.69	252.98	
22	F2	938.14	Calcites (Fs)	− 4.92	− 7.45	126.77	20.47	40.53	113.51	39.43	510.98	
23	F2	945.05	Calcites (Fs)	− 4.13	− 6.03	124.25	23.1	45.37	115.84	32.09	266.36	
24	F3	1234.70	Calcites (Fs)	− 7.12	− 6.17	98.39	17.63	45.72	109.65	32.80	289.88	
25	F4	1244.56	Calcites (Fs)	− 4.39	− 8.17	129.8	17.24	53.89	114.24	43.30	640.14	
26	H3	1568.90	Calcites (Fs)	− 0.79	− 9.65	331.05	147.09	98.01	120.88	51.60	916.51	Group C
27	H6	1349.88	Calcites (Fs)	0.76	− 9.83	342.67	152.28	74.57	123.96	52.63	951.12	
28	H7	1389.67	Calcites (Fs)	− 1.74	− 11.02	296.85	139.08	95.59	118.25	59.66	1185.35	
29	H8	1569.75	Calcites (Fs)	0.79	− 10.88	283.28	165.49	86.43	123.50	58.82	1157.30	
30	F1	780.66	Calcites (Fs)	0.03	− 10.65	307.17	134.25	89.4	122.06	57.45	1111.51	
31	F4	1403.45	Calcites (Fs)	− 1.35	− 9.58	284.26	145.9	90.88	119.76	51.19	903.11	
32	H1	701.36	Calcites (Fs)	− 1.78	− 16.94	410.95	201.58	137.87	115.22	98.83	2490.91	Group D
33	H1	740.28	Calcites (Fs)	− 1.34	− 15.08	454.19	227.72	130.98	117.05	85.77	2055.54	
34	H1	756.46	Silica (Fs)	− 0.45	− 16.32	480.66	296.45	120.16	118.25	94.40	2343.22	
35	H2	1200.36	Silica (Fs)	− 0.69	− 16.57	440.33	274.65	102.9	117.64	96.17	2402.46	
36	H3	1492.88	Silica (Fs)	− 2.43	− 16.34	455.71	339.37	107.16	114.19	94.54	2347.95	
37	H4	1103.79	Silica (Fs)	− 1.9	− 16.7	430.5	306.41	146.33	115.09	97.10	2433.43	
38	H5	842.16	Quartz (Fs)	− 2.72	− 15.22	475.8	233.8	137.21	114.15	86.73	2087.51	
39	H5	897.98	Band mixtures (Fs)	− 0.81	− 16.25	493.52	310.55	125.87	117.55	93.90	2326.71	
40	H5	923.80	Pyroclasts (Fs)	− 2.7	− 16.27	399.34	236.62	88.65	113.67	94.04	2331.42	
41	H5	956.34	Silica (Fs)	− 1.72	− 15.28	413.28	345.92	92.32	116.17	87.14	2101.25	
42	H6	1479.46	Silica (Fs)	− 2.82	− 17.39	390.42	291.68	160.53	112.86	102.09	2599.70	
43	H7	1393.68	Silica (Fs)	− 2.62	− 16.49	431.5	215.28	118.22	113.72	95.60	2383.46	
44	H8	1626.4	Silica (Fs)	0.35	− 16.75	456.64	267.55	108.44	119.68	97.46	2445.38	
45	F1	842.41	Calcites (Fs)	0.26	− 16.84	407.54	231.27	122.65	119.45	98.11	2466.91	
46	F2	1135.28	Silica (Fs)	− 2.82	− 17.1	399.16	313.18	124.64	113.01	99.98	2529.43	
47	F3	1424.93	Silica (Fs)	0.07	− 17.18	390.78	295.09	126.64	118.89	100.56	2548.76	
48	F4	1434.79	Silica (Fs)	− 3.11	− 17.18	382.4	277	128.63	112.38	100.56	2548.76	
aver				− 3.12	− 11.14	286.10	139.89	84.45	115.36	62.41	1277.12	

#### Carbon and oxygen isotopes

The CM and Fs do not show a significant difference in the carbon isotope values. The δ^13^C_PDB_ values of the CM are − 2.88 ‰ to − 1.54 ‰, with an average of − 2.07 ‰ (Table 1). The δ^13^C_PDB_ values of the KF are − 7.70 ‰ to + 0.97 ‰, with an average of − 3.12 ‰ (Table 1).

The oxygen isotope values of CM are much higher than those of Fs. The δ^18^O_PDB_ values of CM are − 6.95 ‰ to − 5.18 ‰, with an average of − 6.10 ‰ (Table 1). The δ^18^O_PDB_ values of Fs are − 17.39 ‰ to − 5.57 ‰, with an average of − 11.14 ‰ (Table 1).

#### Minor elements

**Figure 8:**
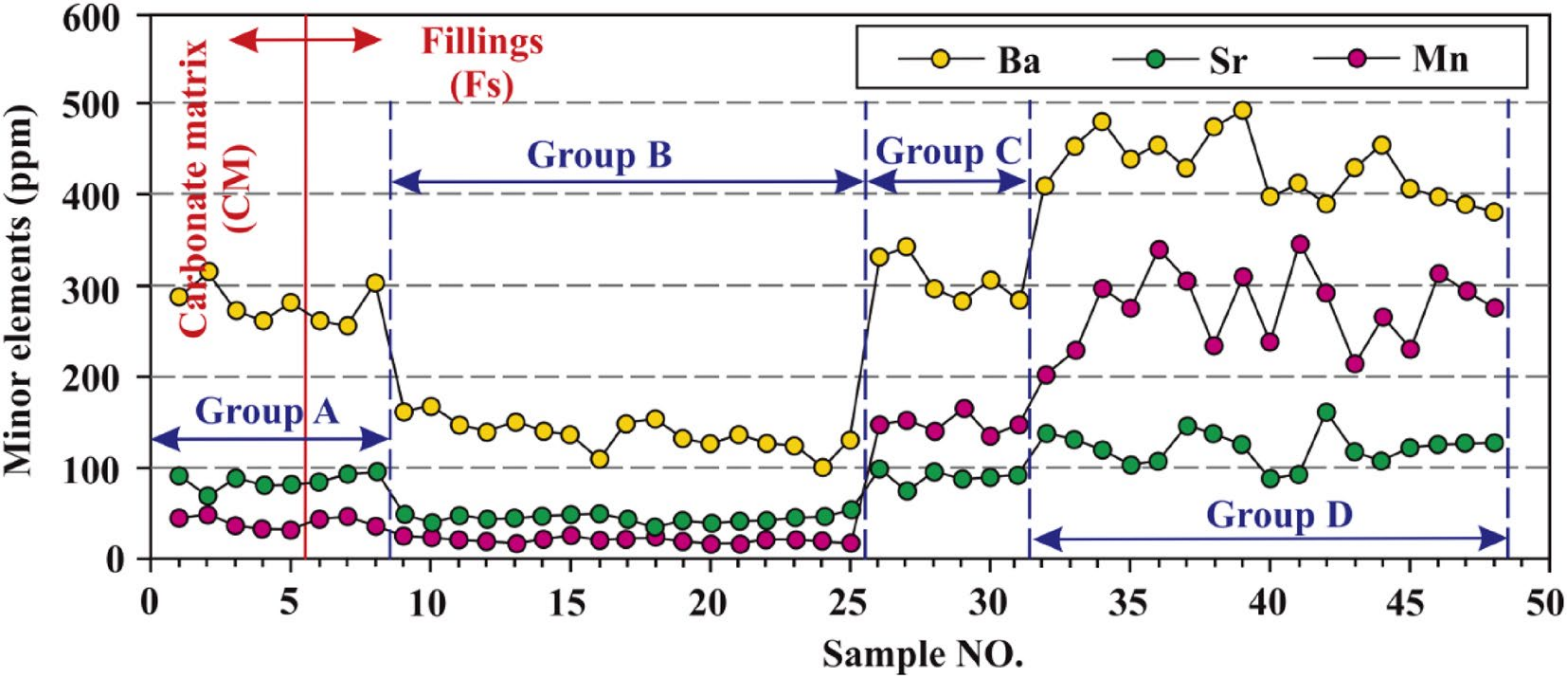
Concentration distribution characteristics of minor elements Ba, Mn, and Sr of the Ordovician carbonate matrix (CM) and fillings (Fs).

In this study, three minor elements, including Ba, Mn, and Sr, were tested and analyzed. The minor element Ba does not differ greatly in CM and Fs, as shown in Fig. 8. The concentrations of Ba in CM are 261.21 ppm to 315.73 ppm, with an average of 284.24 ppm. The concentrations of Ba in Fs are 98.39 ppm to 493.52 ppm, with an average of 286.10 ppm.

The minor element Mn shows significant difference in CM and Fs, as shown in Fig. 8. The concentrations of Mn in CM are 31.67 ppm to 48.28 ppm, with an average of 38.48 ppm (Table 1). The concentrations of Mn in Fs are 15.27 ppm to 345.92 ppm, with an average of 139.89 ppm (Table 1).

The minor element Sr also shows significant difference in CM and Fs, as shown in Fig. 8. The concentrations of Sr in CM are 68.81 ppm to 90.40 ppm, with an average of 81.92 ppm (Table 1). The concentrations of Sr in Fs are 33.26 ppm to 160.53 ppm, with an average of 84.45 ppm (Table 1).

## Discussion

### Formation stages of the Ordovician paleokarst

**Figure 9:**
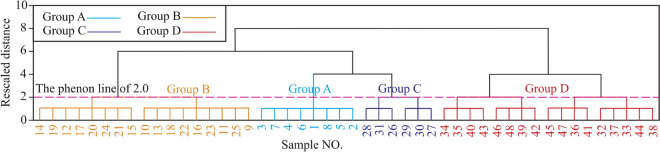
Dendrogram of HCA results.

In general, the oxygen isotope is mainly related to water body salinity and environmental temperature during the formation periods of sediments, while the carbon isotope is primarily affected by evaporation, dynamic fractionation, and early carbonate deposition^7,8,25^. The δ^18^O_PDB_ values of Fs in Table 1 are much smaller than those of CM, suggesting that the Fs formation might be affected by many factors, such as leaching by various waters (e.g. meteoric water, surface water, groundwater), and/or temperature rising, and/or burial depth increasing, and/or hydrothermal (water) activities^7,8,26,27^. Many previous researchers found that the high Ba, Mn, and Sr concentrations in Fs might be related to hydrothermal fluids^4,28–30^, while the low Ba, Mn, and Sr concentrations in Fs might be related to long-term leaching by meteoric water and/or surface water and/or groundwater^30–32^. Therefore, the significant changes of minor elements Ba, Mn, and Sr in Fs (see Fig. 9) suggest that Fs might be formed in different environments.

**Figure 10:**
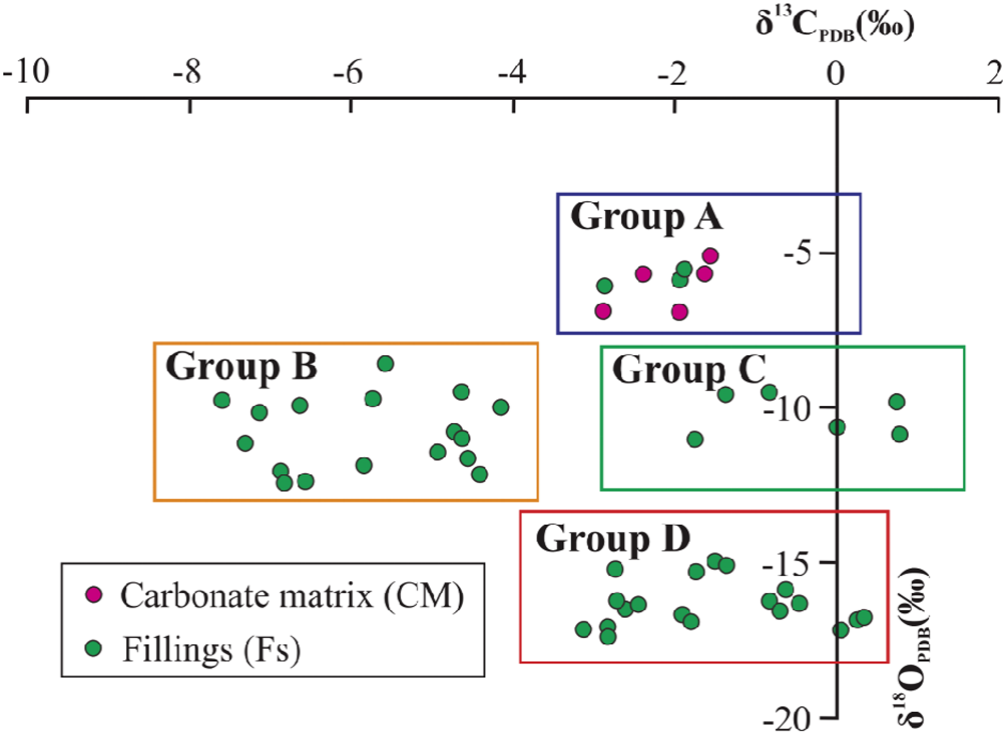
Distribution characteristics of carbon and oxygen isotopes for Groups A, B, C, and D.

Hierarchical cluster analysis (HCA) is one of the most widely applied cluster techniques in geochemical analysis, which can classify similar observations into separate groups to obtain a dendrogram^33,34^. In order to accurately identify the Ordovician paleokarst formation stages in the Huai-Fu Basin, the HCA method was utilized based on the carbon and oxygen isotopes and minor elements Ba, Mn, and Sr. Using the Ward method and Euclidean distance^35,36^, HCA yielded the optimum four groups based on the phenon line of 2.0 (Fig. 9): Group A includes 5 CM and 3 Fs, Group B consists of 17 Fs, Group C has only 6 Fs, Group D includes 17 Fs, the samples of each group are shown in Fig. 9 and Table 1. In consideration of previous results of the Ordovician carbonate paleokarst in Northern China^7,8,37,38^, the following four groups (types) of the Ordovician carbonate paleokarst in the Huai-Fu Basin could be obtained, as shown in Figs. 9 and 10.

Group A: The δ^13^C and δ^18^O values of the Fs samples are similar to those of the CM samples (Fig. 10), and the concentrations of Ba, Mn, and Sr of the Fs samples are also very close to those of the CM samples (Fig. 8), indicating that Fs and CM in Group A might be formed in the same period. In general, the δ^13^C and δ^18^O values of modern marine carbonates are both close to 0 ‰^8,39^, and the δ^13^C and δ^18^O values increase with seawater salinity while decrease with leaching by freshwater and evaporation^7,40^. Therefore, the δ^13^C and δ^18^O values of CM and Fs in Group A are all negative, indicating that they are likely to be developed in shallow burial or near-surface eogenetic environment and have been affected by meteoric water^41,42^.

Group B: The δ^13^C and δ^18^O values of Fs in Group B show a significant negative drift (Fig. 10), and the concentrations of Ba, Mn, and Sr show a sharp drop (Fig. 8), which is the typical characteristics of paleoweathering crust karst^7,8^. From the Late Ordovician to the end of the Early Carboniferous, the Ordovician strata experienced long-term leaching by meteoric water, resulting in a significant negative drift of δ^13^C and δ^18^O in Fs and a large amount migration of trace elements Ba, Mn and Sr^8,43^. Therefore, the δ^13^C, δ^18^O, Ba, Mn, and Sr values of Fs in Group B are apparently much smaller than those of Fs in Group A.

Group C: Relative to CM in Group A, the δ^13^C values in this group show a slight positive drift while the δ^18^O values show a slight negative drift (Fig. 10), which is a typical characteristic of mesogenetic karst (pressure-released water karst)^44^. In the deep-buried karst area, organic materials were decomposed and methylated under methanogenic conditions, making the δ^13^C values drifted positively^8^. The concentrations of Ba and Sr in Group C show a slight increase and the concentration of Mn shows a significant increase (Fig. 8), suggesting that these minerals might come from the overlying coal-bearing formations during diagenetic compaction^30^.

Group D: A large number of studies have shown that when the δ^18^O values of Fs in the Cambrian and Ordovician karst are less than − 12 ‰, Fs was most likely formed by hydrothermal (water) activities^4,29,30^. The δ^18^O in the hydrothermal (water) undergo a heat loss during the transformation^28,30^, causing the δ^18^O values of Fs in Group D becoming much smaller than those in Groups A, B, and C, as shown in Fig. 10. Fs in Group D has high concentrations of Ba, Mn, and Sr (Fig. 8), which is a typical characteristic of hydrothermal fillings^28,30^.

### Formation environments of the Ordovician paleokarst

The carbon and oxygen isotopes are important geochemical tracers, which could provide important information of the paleokarst formation environments, such as paleosalinity, paleotemperature, paleodepth, etc., thus they were often used to estimate the formation environments of multistage paleokarst^7,8,30,45–47^.

#### Calculation of paleosalinity, paleotemperature, and paleodepth

Keith and Webber^48^ proposed an empirical formula for calculating paleosalinity (*Z*) based on the values of δ^13^C_PDB_ and δ^18^O_PDB_ isotopes, which have been used to distinguish between continental and marine sedimentary environments. The empirical formula and the criterion are as follows:

1 $$ Z = 2.048 \times \left( {\delta^{13} C_{PDB} + 50} \right) + 0.498 \times \left( {\delta^{18} O_{PDB} + 50} \right) $$

When Z > 120, it is marine sediments, and when Z < 120, it is freshwater sediments^48^. When Z is close to 120, the sediments may be affected by both marine water and freshwater^48^. This empirical formula and criterion have been widely used worldwide^5,48,49^.

Urey^50^ proposed a method for calculating the paleo-ocean temperature (*T*) based on the values of δ^18^O_PDB_ isotope, which was further elaborated by Epstein and Mayeda^51^. Zhang et al.^7^ corrected the time effect in the formula proposed by Urey^50^ based on the history of geological evolution in China. The improved formula proposed by Zhang et al.^7^ has been widely used in China^2,7,8^, so it was also employed in this study. The improved formula for calculating the paleotemperature (*T*) is as follows:

2 $$ T = 16.9 - 4.38 \times (\delta_{18} O + 2.8) + 0.1 \times (\delta_{18} O + 2.8)^{2} $$

Assuming that the surface paleotemperature in the Huai-Fu Basin is 16.8 °C, the paleodepth of constant temperature zone is 30 m, and the paleo-geothermal gradient is 30 °C /km as adopted by many researches^52^, then the formula for calculating the paleodepth (*H*) of the formation of the Ordovician paleokarst is:

3 $$ H = \frac{(T - 25) \times 1000}{{30}} + 30 $$

In summary, the *Z*, *T*, and *H* values of the formation of the Ordovician paleokarst in the Huai-Fu Basin can be calculated using Eqs. (1), (2), and (3), respectively, and the results are shown in Table 1.

#### Analysis of paleokarst formation environments

**Figure 11:**
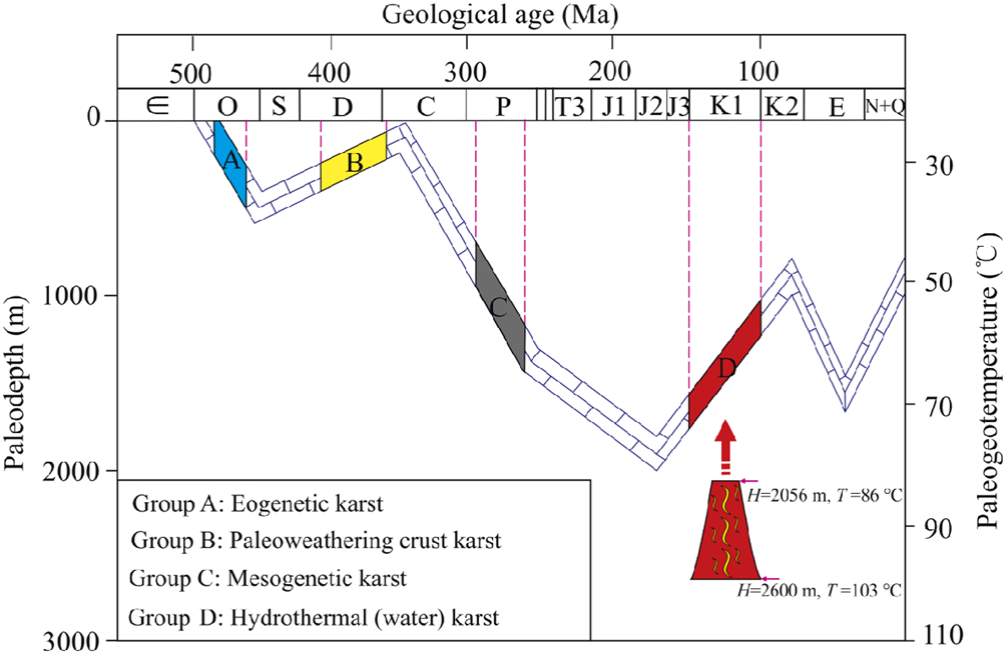
Formation environments of the Ordovician carbonate paleokarst in the Southern North China Basin.

According to Table 1 and the buried history of the Ordovician strata in the Huai-Fu Basin, the formation ages and environments of the four groups paleokarst in the Ordovician carbonates could be obtained, as shown in Fig. 11 and Table 2.

**Table 2:** Paleosalinity, paleotemperature, and paleodepth of the formation of the Ordovician carbonate paleokarst in Groups A, B, C, and D.

Clustering groups	Carbon and oxygen isotopes		Paleosalinity, paleotemperature and paleodepth		
	δ^13^C_PDB_ (‰)	δ^18^O_PDB_ (‰)	Paleosalinity *Z*	Paleotemperature *T* (°C)	Paleodepth *H* (m)
	min to max/aver	min to max/aver	min to max/aver	min to max/aver	min to max/aver
Group A	− 2.88 to − 1.54/− 2.13	− 6.95 to − 5.18/− 6.02	117.96 to 121.57/119.95	27.89 to 36.80/32.05	126.36 to 423.31/265.00
Group B	− 7.70 to − 4.13/− 5.77	− 8.45 to − 5.72/− 7.05	108.66 to 115.84/111.98	30.54 to 44.84/37.38	214.74 to 691.31/442.78
Group C	− 1.74 to 0.79/− 0.38	− 11.02 to − 9.58/− 10.27	118.25 to 123.96/121.40	51.19 to 59.66/55.22	903.11 to 1185.35/1037.48
Group D	− 3.11 to 0.35/− 1.60	− 17.39 to − 15.08/− 16.46	112.38 to 119.68/115.82	85.77 to 102.09/ 95.47	2055.54 to 2599.70/2378.99

Group A may represent an eogenetic karst stage, which might be developed from the Middle to Late Ordovician (Fig. 11) according to the *Z*, *T*, and *H* values of Group A in Table 2. The *Z* values range from 117.96 to 121.57, with an average of 119.95 (Table 2), indicating that the formation of eogenetic karst might be affected by both marine water and meteoric water^48^. Moreover, the *T* values in Group A range from 27.89 to 36.80 °C, with an average of 32.05 °C (Table 2), and the *H* values range from 126.36 to 423.31 m, with an average of 265.0 m (Table 2), indicating that the eogenetic karst might be developed in a relatively open and shallow buried environment.

Group B may represent a paleoweathering crust karst stage, which might be developed from the Middle Devonian to Early Carboniferous (Fig. 11) according to the *Z*, *T*, and *H* values of Group B in Table 2. The *Z* values range from 108.66 to 115.84, with an average of 111.98 (Table 2), indicating the formation of paleoweathering crust karst primarily being affected by freshwater (e.g. meteoric water, surface water, and groundwater)^53,54^. The *T* values in Group B range from 30.54 to 44.84 °C, with an average of 37.38 °C (Table 2), and the *H* values range from 214.74 to 691.31 m, with an average of 442.78 m (Table 2). These data indicate that the paleoweathering crust karst was developed in a near-surface and shallow burial environment, and its formation was also affected by meteoric water, surface water and groundwater^8,37,54^.

Group C may represent a mesogenetic karst stage (pressure-released water karst stage), which might be developed during the Permian (Fig. 11) according to the *Z*, *T*, and *H* values of Group C in Table 2. The *Z* values range from 118.25 to 123.96 (Table 2), with an average of 121.4 (> 120), which might be related to the high salinity brines released by the Carboniferous-Permian in a compacted diagenetic environment^8^. Brines could penetrate into the underlying Ordovician carbonates through pores (vugs), fractures, bedding, and paleoweathering crust, forming highly salty karst Fs^44^. The *T* values in Group C range from 51.19 to 59.66 °C, with an average of 55.22 °C, and the *H* values range from 903.11 to 1185.35 m, with an average of 1037.48 m, indicating that the mesogenetic karst was formed in a closed and deep buried environment with medium–high temperature.

Group D may represent a hydrothermal (water) karst stage, which might be developed in the Early Cretaceous (Fig. 11) combined with the results of Xu et al.^20^. The related magmatic intrusions that occurred in this period are widely distributed in the Paleozoic and Mesozoic strata. As shown in Table 2, the *T* values in Group D range from 85.77 to 102.09 °C, with an average of 95.47 °C, and the *H* ranges from 2055.54 to 2599.70 m, with an average of 2378.99 m. The *T* and *H* values in Group D exceed the maximum temperature (< 85 °C) and maximum burial depth (< 2000 m) of the Ordovician strata, respectively, as shown in Fig. 11, suggesting that the hydrothermal fluids (waters) must come from a much deeper depth. The *Z* values in Group D range from 112.38 to 119.68, with an average of 115.82 (Table 2), indicating that hydrothermal activities might be accompanied by groundwater and/or surface water activities^7,9,42^.

### Formation mechanisms of the Ordovician paleokarst

#### Syngenetic karst

Since the fillings of the selective dissolution pores are difficult to obtain, syngenetic karst could not be identified by the carbon and oxygen isotopes and minor elements Ba, Mn, and Sr, but we have successfully identified syngenetic karst from pores and fillings characteristics. Many selective dissolution pores, such as biofilm pores (Fig. 5a), gypsum pores (Fig. 5b), and geopetal structure (Fig. 5c), which have been considered as signs of syngenetic karst^38,55^, were all found in the Ordovician carbonate rocks in the Huai-Fu Basin.

**Figure 12:**
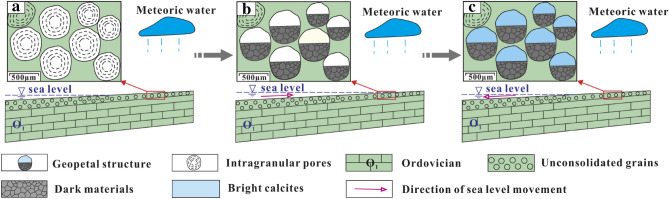
Development model of geopetal structure: (**a**) Intragranular pores formation; (**b**) Dark materials was deposited at the bottom of the intragranular pores; (**c**) Bright calcites was deposited on the upper parts of the dark materials.

Syngenetic karst, also known as syn-depositional karst, refers to the selective dissolution of unstable minerals (such as aragonite, high-magnesium calcite, etc.) by meteoric water during short-term exposure as a result of syn-sedimentary sea-level change at sediment deposition^38^. Syngenetic karst generally takes place where the sediments have reached or exceeded sea level. Gypsum nodules that are more susceptible to dissolution were first selectively dissolved due to the leaching by meteoric water, forming gypsum dissolution pores (Fig. 5b). Then, carbonate grains at the top and margin of the shoal are also influenced by meteoric water, forming intragranular pores, as shown in Fig. 12a. When the sea level rose, a layer of dark materials was first deposited at the bottom of some intragranular dissolution pores, as shown in Fig. 12b; then when the sea level declined, the upper parts of dark materials would be covered by a layer of bright calcites, as shown in Fig. 12c. This pore-filling structure in which the upper part was filled with bright calcites while the lower part was filled with dark materials was called geopetal structure^23^. The selective dissolution processes were repeated until the sediments entered the shallow burial stage.

#### Eogenetic karst

Eogenetic karst was developed in a shallow burial or near-surface eogenetic environment^56^, and characterized by the development of intercrystalline pores (Fig. 5d), intergranular pores (Fig. 5e), dissolved fractures (Fig. 5b), and small dissolution channels (Fig. 5f), but they are difficult to completely preserve them due to the later alteration.

**Figure 13:**
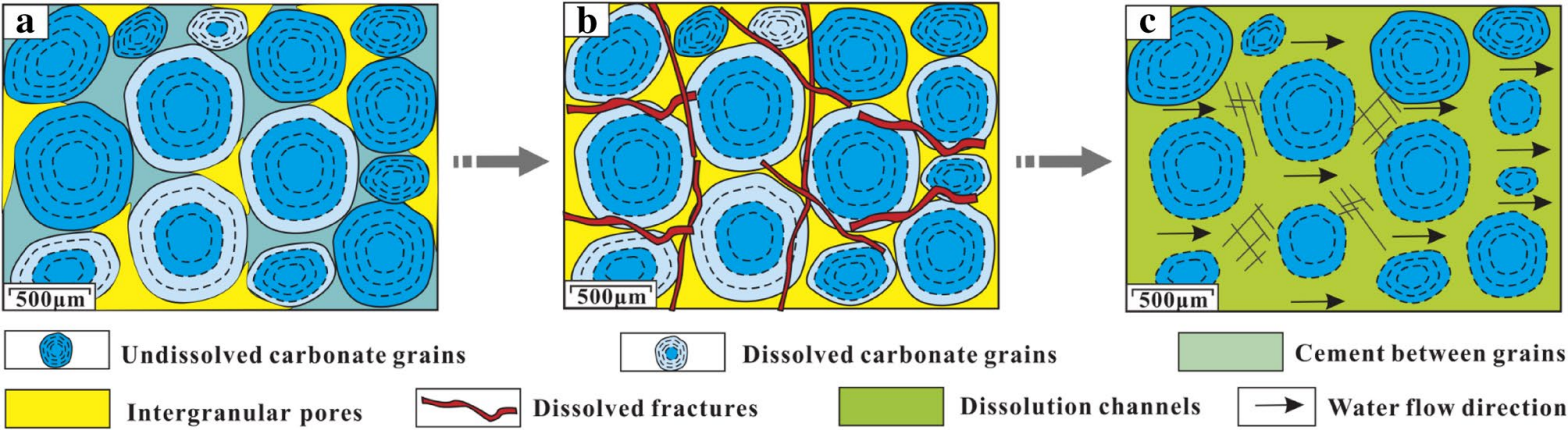
Development model of small dissolution channels: (**a**) Intercrystalline and intergranular dissolution pores formation; (**b**) Dissolution fractures formation; (**c**) Small dissolution channels formation.

After a period of deposition, the Ordovician sediments began to enter the burial diagenesis stage. Due to the shallow burial (*H* = 126.36 to 423.31 m, see Table 2) and weak compaction of sediments during the early diagenesis, cementation between the carbonate grains was weak^57–59^. Co-affected by marine water and meteoric water (*Z* = 117.96 to 121.57, see Table 2), water flew along the weak cementation surfaces and continuously dissolved the cement, thereby forming intercrystalline and intergranular dissolution pores, as shown in Fig. 13a. Then, as the sea level gradually declined and the land gradually uplifted, the intercrystalline and intergranular pores continued to dissolve and expand in the CO_2_-rich meteoric water^38^, forming dissolved fractures, as shown in Fig. 13b. Over time, dissolution accelerated with increasing water flow, and then small dissolution channels were formed^24^, as shown in Fig. 13c. The above process was repeated until these pores, early fractures, and small channels were filled with calcites^38,58,60^.

Eogenetic karst was also developed in the Ordovician carbonates in the Ordos Basin and Tarim Basin, where the measured values of carbon, oxygen isotopes and the minor elements Ba, Mn, and Sr of Fs formed in an eogenetic environment were very close to that of the carbonate matrix^8,38^, and the *Z* values are also around 120^8,38^. These characteristics are very similar to the eogenetic karst in Ordovician carbonates in the Huai-Fu Basin.

#### Paleoweathering crust karst

Paleoweathering crust karst was commonly developed within 0–100 m below the Ordovician unconformities in Northern China^53,54^. It was characterized by the development of dissolution vugs, high-angle fractures, channels, and caves, and the channels and caves were generally filled with karst breccias and gray-green argillaceous muds.

**Figure 14:**
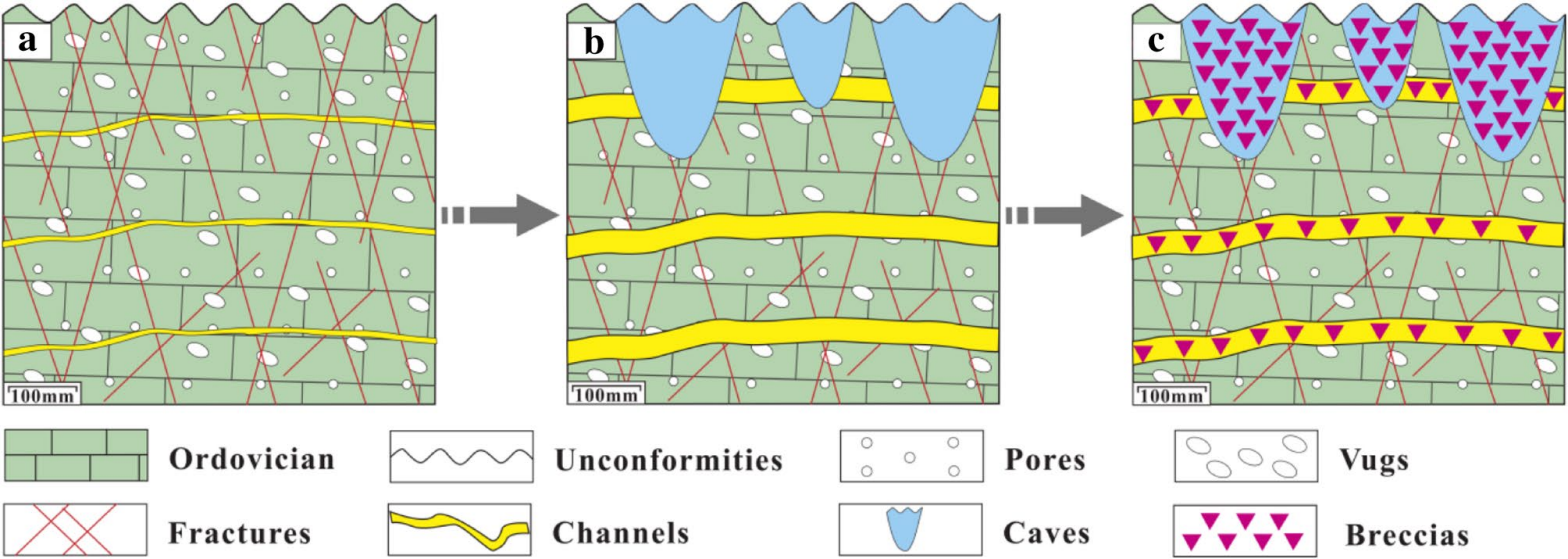
Development model of caves and channels in the Ordovician paleoweathering crust: (**a**) A porous geological body formation; (**b**) Large dissolution channels and caves formation; (**c**) Collapse breccias filled the channels and caves.

Affected by the Caledonian movement, uplifts generally occurred in Northern China after the Late Ordovician^38^, resulting in the Ordovician carbonates completely exposed to weathering and erosion environments of the surface or near-surface (*Z* = 108.66 to 115.84, *T* = 30.54 to 44.84 °C, see Table 2). Therefore, a large number of dissolution pores, vugs, and fractures, especially high-angle fractures were first developed at the top of the Ordovician strata^53^. Over time, dissolution was strengthened with increasing water flow. Consequently, most of the dissolved fractures and small dissolution channels formed during the eogenetic karst stage were revived. These irregular pores, vugs, fractures, and small channels were spatially inter-connected, forming a porous geological body composed of pores, vugs, fractures, and small channels, which showed spongy dissolution characteristics in a cross-sectional view (Fig. 14a)^46,61^. With the further dissolution of the spongy body, the structure of the carbonate matrix was destroyed, and large dissolution channels and caves were formed and connected through vugs and fractures (Fig. 14b). In such a context, breccias were formed if the overlying or surrounding rocks lost support under the action of gravity and/or mechanical erosion and/or chemical dissolution^62^, filling the channels and caves (Fig. 14c).

At the end of the Middle Carboniferous, the North China Plate began to sink and received deposition again^42,54^. The gray-green muds from the Carboniferous Benxi Formation first covered over the Ordovician paleoweathering crust and filled in the channels and caves through fractures. Except for gray-green muds, pores (including vugs, fractures, caves, and channels) in paleoweathering crust were also filled with calcites due to the long-term dissolution of meteoric water, surface water, and groundwater.

#### Mesogenetic karst

Mesogenetic karst, also known as pressure-released water karst, refers to some corrosive components generated from hydrocarbon-generating formations under the diagenetic compaction, which corroded and dissolved carbonate rocks^63,64^.

**Figure 15:**
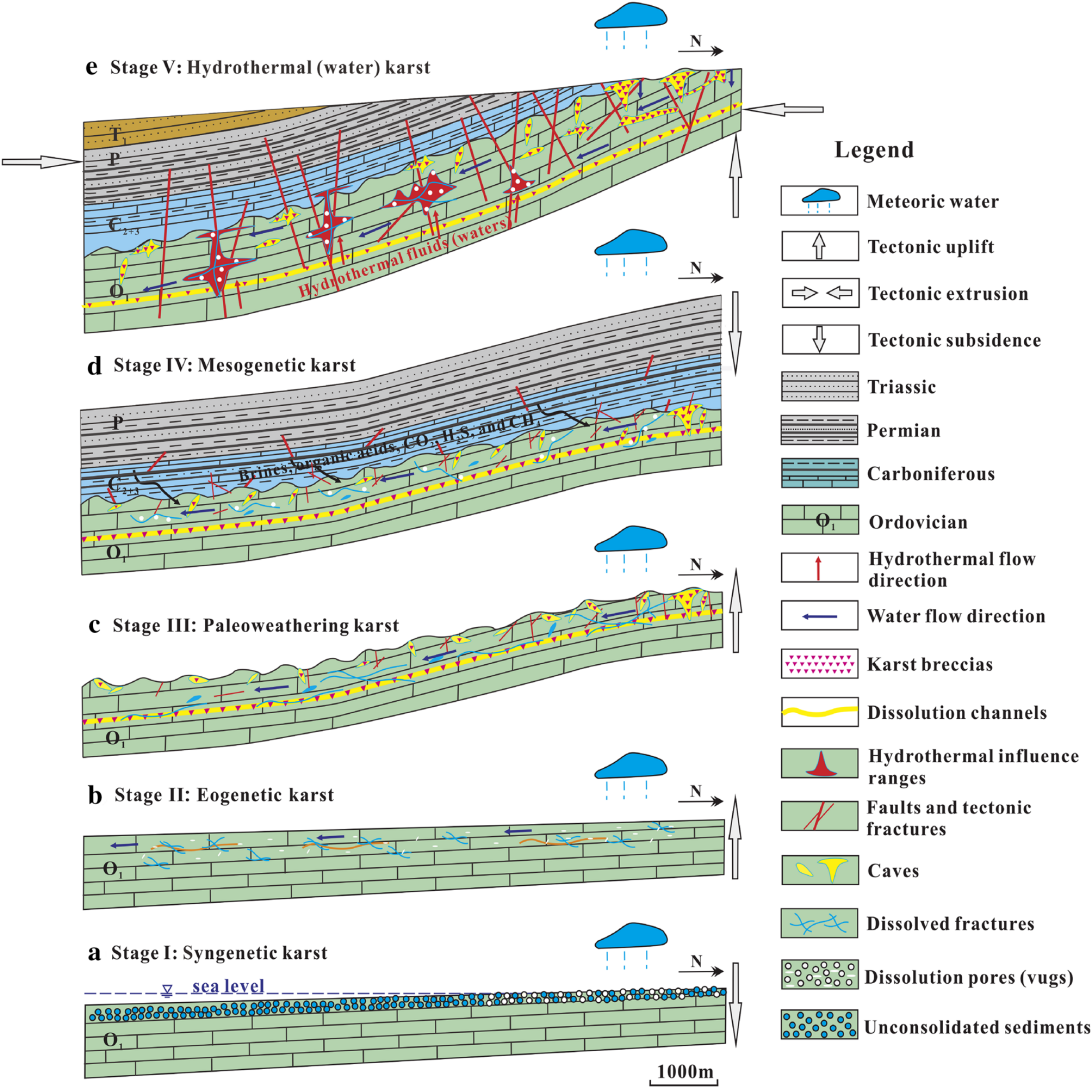
Evolution model of the Ordovician paleokarst in the Southern North China Basin: (**a**) Syngenetic karst formation stage; (**b**) Eogenetic karst formation stage; (**c**) Paleoweathering karst formation stage; (**d**) Mesogenetic karst formation stage; (**e**) Hydrothermal (water) karst formation stage. Maps created by the authors with CorelDRAW Graphics Suite X7 (https://www.coreldraw.com/en/?link=wm).

At the end of the Middle Carboniferous, affected by the Hercynian movement, the Northern China platform declined overall and started to receive deposition^65^. Covered by the Carboniferous-Permian strata, the Ordovician strata became a relatively deep closed environment. Driven by the pressure difference, brines, organic acids, CO_2_, H_2_S, and CH_4_ produced by the diagenesis of organic matter and thermochemical sulfate reduction (TSR) of the Permian-Carboniferous strata were continuously squeezed out^15,63,66,67^. These corrosive components penetrated into the Ordovician carbonate rocks through vugs, fractures, bedding planes, etc., producing a large number of new pores and fractures, as shown in Fig. 15d. Thus, we found that some organic matters remained in dissolution pores (Fig. 5m), fractures (Fig. 5j), and stylolites (Fig. 5k).

The mesogenetic karst was also common in the Lower Paleozoic Cambrian and Ordovician carbonates in the Tarim Basin and Ordos Basin^8,30,56^, showing that the δ^13^C values of fillings had a slight positive drift while the δ^18^O values had a slight negative drift^7,8^, which is very similar to the mesogenetic karst in the Ordovician carbonates in the Huai-Fu Basin, as shown in Fig. 10. Mazzullo and Harris^64^ showed that the δ^13^C values of fillings formed during mesogenetic karst stage exhibited a positive drift, which could be attributed to the decomposition and methanation (CH_4_) of organic matter caused by methane bacteria. The CH_4_ formed during biochemical processes was rich in δ^12^C whereas the CO_2_ had abundant δ^13^C^8^, thus the cement fillings of the mesogenetic karst were enriched in δ^13^ C, showing a slight positive drift^44,64^. The δ^18^O values of fillings in Group C exhibited a slight negative drift, as shown in Fig. 10, which could be related to that the mesogenetic karst was formed in a relatively deep depth (*H* = 903.11 to 1185.35 m, see Table 2) and a relatively high temperature (*T* = 51.19 to 59.66 °C, see Table 2), because the δ^18^O values would decrease with increasing depth and temperature^7,8^.

#### Hydrothermal (water) karst

Hydrothermal minerals (e.g. silica, flints, quartz, pyroclasts, chalcedonies, and hydrothermal dolomites, see Fig. 5g,l,m,n,o) were discovered in pores, vugs, and fractures in the Ordovician carbonates in the Huai-Fu Basin, and the δ^18^O values of these hydrothermal minerals were less than − 12 ‰ (Table 1), both indicating that the formation of this kind of karst was related to hydrothermal activities^4,5,29,30,67,68^. Moreover, some calcites were also found around the hydrothermal minerals (Fig. 5l), and the δ^18^O values of these calcites were also less than − 12‰ (Table 1), indicating that hydrothermal activities might be accompanied by groundwater and/or surface water activities. Therefore, the origin of these minerals and calcites were primarily related to hydrothermal fluids (waters) – rock interaction, called hydrothermal (water) karst^8^.

The hydrothermal minerals and calcites both have lower negative δ^18^O values, and their formation depth exceeds 2000 m (Table 2) and temperature exceeds 85 °C (Table 2), indicating a deep burial fluid origin. What is more, further observations showed that these hydrothermal minerals and calcites were mainly distributed in pores, vugs, and fractures near some deep fault and fold zones, suggesting that their formations may be related to faults and fractures. This phenomenon was also found in the Lower Paleozoic carbonates in the major oil and gas basins in Northern China, such as the Tarim Basin^6,63^, the Ordos Basin^13^, and the Bohai Bay Basin^11,69^, suggesting that faults and fractures were the channels for the hydrothermal fluids and waters transport^30,42,63^.

The rooted faults and their fault-related fracture network served as channels for the hydrothermal fluids and groundwater to move upward, as shown in Fig. 15e, and they connected deep materials with sedimentary formations through thermal convection^4,6,67^. In general, deep hydrothermal fluids (waters) generally have high temperatures and high concentrations of CO_2_ and H_2_S produced by the thermochemical sulphate reduction (TSR)^4,30,42^. During upward migration, the hydrothermal fluids (waters) continuously dissolved carbonate rocks and formed a large number of new pores, vugs, and fractures, which were then filled by hydrothermal crystals (such as silica, flints, quartz, pyroclasts, chalcedonies, etc.) and calcites.

### Evolution model of the Ordovician paleokarst

Based on the stratigraphic sedimentary and tectonic evolution history of the Huai-Fu Basin, combined with the analysis on the formation environments and mechanisms of the Ordovician carbonate paleokarst, an evolution model of the Ordovician multistage paleokarst in the study area was established, which includes the following five stages, as shown in Fig. 15.

*Stage I*: The Ordovician carbonates in the Huai-Fu Basin were deposited primarily in a tidal flat environment. Syngenetic karst took place when sediments were exposed to the atmosphere due to the intermittent drop of sea level. The unstable minerals in the sediments were first dissolved by meteoric water, forming selective dissolution pores (Fig. 15a), such as biofilm pores, gypsum pores, intragranular pores, and geopetal structure^38^. However, the development of such pores was restricted due to the limited subaerial exposure area and the long time of being under the sea level, and most of these pores were cemented by calcites (Fig. 5a–c), thus they were less important for carbonate reservoirs in the Huai-Fu Basin.

*Stage II*: During the Middle and Late Ordovician, the Ordovician strata in the Huai-Fu Basin entered into a shallow-burial eogenetic environment. When the sea level gradually fell, CO_2_-rich meteoric water percolated into the loose carbonate of the Ordovician strata and flowed along the weak cementation surfaces (Fig. 15b). Then, a large scale of non-selective dissolution took place, forming a great number of intercrystalline pores (Fig. 5c), intergranular pores (Fig. 5d), dissolved fractures (Fig. 5b), and small dissolution channels (Fig. 5f). However, like the pores formed in the syngenetic karst stage, most of these pores formed in the eogenetic karst stage were altered or filled in diagenetic alteration^6,42^, thus they were also less important for carbonate reservoirs in the Huai-Fu Basin.

*Stage III*: Affected by the Caledonian movement, uplifts generally occurred in Northern China in the Late Ordovician, lasting about 120 Ma^38^. The Ordovician strata was exposed to the surface or near-surface, and was weathered, eroded, and dissolved by the mixing water (meteoric water, surface water, and groundwater). Many high-angle fractures (Fig. 7a), vugs (Fig. 3a,d,e,f), caves (Fig. 7a), and channels (Fig. 6a,b) were formed within 0 – 30 m below the Ordovician unconformities in the Huai-Fu Basin (Fig. 15c), which were partially filled with karst breccias and gray-green argillaceous muds or unfilled. Field tests showed that the porosity of the paleoweathering crust karst ranges from 5.12% to 34.16%, with an average of 22.13%, and the permeability is 102–1960 mini Darcy (mD). Therefore, the Ordovician paleoweathering crust with high porosity and high permeability is the focus of oil and gas exploration in the Huai-Fu Basin and other Basins in Northern China^54^.

*Stage IV*: During the Permian period, the Ordovician strata entered into a relatively deep closed environment. Driven by the pressure difference, acidic materials (such as organic acids, carbon dioxide, hydrogen sulfide, methane, etc.) were produced by the diagenesis of organic matter and TSR of the Permian-Carboniferous strata^15,66,67^. Such acidic materials penetrated into the underlying Ordovician carbonates and created some new pores and fractures (Fig. 15d), forming mesogenetic karst, also known as an organic acid and compacted-released water karst^8,63,64^. However, most of these new pores and fractures were almost completely filled with organic matters and/or calcites, as shown in Fig. 5f,j,k. Therefore, the mesogenetic karst may have little contribution to the Ordovician carbonate reservoirs in the Huai-Fu Basin.

*Stage V*: Hydrothermal (water) karst primarily occurred in the Early Cretaceous in the Huai-Fu Basin. The hydrothermal fluids (waters) with high temperatures and high concentrations of CO_2_ and H_2_S moved upward through some deep faults and fractures and dissolved carbonate rocks (Fig. 15e), forming a large number of new pores, vugs, and fractures. Thus, many previous investigators had emphasized that hydrothermal activities might have a great contribution to porosity and permeability enhancement^4,5,67^. However, most of the new pores, vugs, and fractures created by hydrothermal (water) activities were full-filled with hydrothermal crystals (such as silica, flints, quartz, pyroclasts, chalcedony, etc.) and calcites, as shown in Fig. 5g,l,m,n,o. Therefore, we concluded that the hydrothermal (water) karst may not contribute too much to porosity enhancement of the Ordovician carbonate reservoir in the Huai-Fu Basin. Instead, the hydrothermal (water) karst may make a great contribution to the underlying Cambrian carbonate reservoirs, because the hydrothermal karst in the Cambrian carbonates was mostly semi-filled, and the hydrothermal vug-fracture-cave systems were well developed locally. The Cambrian strata are below the Ordovician strata in the Huai-Fu Basin, with a thickness of more than 1000 m. When the hydrothermal fluids (waters) flew upward from the depth, the dissolution capacity gradually decreased from the deep Cambrian to the shallow Ordovician, while the effects of filling and cementation were enhanced^30^. Therefore, the porosity created by hydrothermal (water) activities may consist of a vital carbonate reservoir space for the Cambrian strata in the Huai-Fu Basin. This phenomenon has also been reported in some areas of Northern China. For example, Zhang et al.^6^ found that the hydrothermal fluids contributed on porosity occlusions of the Ordovician carbonate reservoirs in northwestern Tazhong condensate field, but might have a great contribution to porosity enhancements of the Cambrian carbonate reservoirs.

## Conclusions

The following conclusions can be made from this study.

1. Intragranular pores, intercrystalline (intergranular) pores, dissolution pores (vugs), fractures, channels, and caves are the major pore types in the Ordovician carbonate rocks in the Huai-Fu Basin of China. Pores, vugs, and fractures are mostly filled with calcites, and/or organic matters, and/or thermal minerals, and channels and caves are usually with karst breccias and/or muds, but some vugs, fractures, channels, and caves developed in the fault and fold zones and Ordovician paleoweathering crust are usually unfilled or semi-filled.
2. Five types and five formation environments of the Ordovician carbonate paleokarst have been identified in the Huai-Fu Basin based on paleokarst morphology and geochemical characteristics, including a syngenetic karst formed in a short-term exposure syn-depositional environment, an eogenetic karst formed in a shallow burial or near-surface eogenetic environment, a paleoweathering crust karst formed in an open environment near the surface, a mesogenetic karst formed in a closed buried compacted diagenetic environment, and a hydrothermal (water) karst formed in a deep-burial high-temperature environment.
3. Meteoric water leaching and weak cementation of carbonate grains are the main reasons for the development of dissolution pores of the syngenetic karst and eogenetic karst. The continuous crustal uplift and the combined erosion and dissolution of meteoric water, surface water and groundwater are the main controlling factors for the formation of the paleoweathering crust karst. Brines, organic acids, CO_2_, H_2_S, and CH_4_ produced by the diagenesis of organic matter and thermochemical sulfate reduction (TSR) of the Permian-Carboniferous strata are the main reasons for mesogenetic karst creation. Hydrothermal fluids (waters) from deep depth migrate upward through structures such as faults and fractures to dissolve carbonate rocks, forming the hydrothermal (water) karst formation.
4. High-angle fractures, vugs, caves, and channels are well developed in the Ordovician paleoweathering crust karst, forming a porous geological body with the porosity of 5.12% to 34.16% and the permeability of 102–1960 mD. Therefore, the Ordovician paleoweathering crust with a thick cover and closed environment is the primary target for the exploration of oil and gas reservoirs in the Huai-Fu Basin.
5. Most of the dissolution pores or/and fractures formed in the syngenetic karst, eogenetic karst, mesogenetic karst, and hydrothermal (water) karst stages are almost full-filled, thus they are less important for the Ordovician carbonate reservoirs in the Huai-Fu Basin. However, the pores (e.g. vugs and fractures) created by hydrothermal (water) activities may occupy most of the carbonate reservoir porosity space in the Cambrian strata, which may become another important target for further oil and gas exploration in the Huai-Fu Basin and even in Southern North China Basin.

## References

[1] Liu X (2017). A comparative study of salient petroleum features of the Proterozoic Lower Paleozoic succession in major petroliferous basins in the world. Energy Explor. Exploit..

[2] Sun G (2015). Diagenesis and sedimentary environment of Miocene series in Eboliang III area. Environ. Earth Sci..

[3] Xia L-W, Cao J, Wang M, Mi J-L, Wang T-T (2019). A review of carbonates as hydrocarbon source rocks: basic geochemistry and oil–gas generation. Petrol. Sci..

[4] Cai C, Li K, Li H, Zhang B (2008). Evidence for cross formational hot brine flow from integrated 87Sr/86Sr, REE and fluid inclusions of the Ordovician veins in Central Tarim, China. Appl. Geochem..

[5] Jiang Y (2015). Characteristics and origin of tuff-type tight oil in Jimusaer sag, Junggar Basin, NW China. Petrol. Explor. Dev..

[6] Zhang H, Cai Z, Qi L, Yun L (2017). Diagenesis and origin of porosity formation of Upper Ordovician carbonate reservoir in northwestern Tazhong condensate field. J. Nat. Gas Sci. Eng..

[7] Zhang Q (2016). Environmental and geochemical significance of carbon and oxygen isotopes of Ordovician carbonate paleokarst in Lunnan, Tarim Basin. Environ. Earth Sci..

[8] Zhang Q (2018). Characteristics of Ordovician paleokarst inclusions and their implications for paleoenvironmental and geological history in Halahatang area of northern Tarim Basin. Carbonates Evaporites.

[9] Qing H, Chi G, Zhang S (2006). Origin of coarse-crystalline calcite cement in Early Ordovician carbonate rocks, Ordos basin, northern China: Insights from oxygen and carbon isotopes and fluid inclusion microthermometry. J. Geochem. Explor..

[10] Jiang Y-L, Fang L, Liu J-D, Hu H-J, Xu T-W (2016). Hydrocarbon charge history of the Paleogene reservoir in the northern Dongpu Depression, Bohai Bay Basin, China. Petrol. Sci..

[11] Jin Q, Mao J, Du Y, Huang X (2015). Fracture filling mechanisms in the carbonate buried-hill of Futai Oilfield in Bohai Bay Basin, East China. Petrol. Explor. Dev..

[12] Ren C, Gao X, Jiang H, Li J, He F (2018). Characteristics and favorable area prediction of Ordovician buried-hill carbonate reservoirs in the Bozhong 21–2 tectonic belt, Bohai Bay Basin, China. Petrol. Sci. Technol..

[13] Zhao W (2014). The porosity origin of dolostone reservoirs in the Tarim, Sichuan and Ordos basins and its implication to reservoir prediction. Sci. China Earth Sci..

[14] Huang B, Zhu R, Otofuji Y, Yang Z (2000). The Early Paleozoic paleogeography of the North China block and the other major blocks of China. Chin. Sci. Bull..

[15] Zhao W (2009). Relationship between the later strong gas-charging and the improvement of the reservoir capacity in deep Ordovician carbonate reservoir in Tazhong area, Tarim Basin. Chin. Sci. Bull..

[16] Shu L, Yin H, Faure M, Chen Y (2017). Mesozoic intracontinental underthrust in the SE margin of the North China Block: Insights from the Xu-Huai thrust-and-fold belt. J. Asian Earth Sci..

[17] Zhang KJ (1997). North and South China collision along the eastern and southern North China margins. Tectonophysics.

[18] Zhang H (2005). Evolution of the CBM reservoir-forming dynamic system with mixed secondary biogenic and thermogenic gases in the Huainan Coalfield, China. Chin. Sci. Bull..

[19] Chen S, Wu D, Liu G, Sun R (2017). Raman spectral characteristics of magmatic-contact metamorphic coals from Huainan Coalfield, China. Spectrochim. Acta Part A Mol. Biomol. Spectrosc..

[20] Xu WL, Qinghai W, Xiaochun LIU, Dongyan W, Jinghui GUO (2004). Chronology and Sources of Mesozoic Intrusive Complexes in the Xuzhou-Huainan Region, Central China: Constraints from SHRIMP Zircon U-Pb Dating. Acta Geol. Sin..

[21] Wang Q, Li W, Liu Q (2019). Geological composition and structure of the filling zone and its water-resisting property evaluation on the top of Ordovician limestone. Geofluids.

[22] Shukla MK, Sharma A (2018). A brief review on breccia: It's contrasting origin and diagnostic signatures. Solid Earth Sci..

[23] Rehfisch MW, Webb JA (1993). The Early Devonian Coopers Creek Limestone: A deep-water redeposited limestone in the Melbourne Trough, southeastern Australia. Aust. J. Earth Sci..

[24] Vacher HL, Mylroie JE (2002). Eogenetic karst from the perspective of an equivalent porous medium. Carbonates Evaporites.

[25] Lachniet MS (2009). Climatic and environmental controls on speleothem oxygen-isotope values. Quatern. Sci. Rev..

[26] Maheshwari A, Sial AN, Chittora VK (1999). High-δ13C Paleoproterozoic Carbonates from the Aravalli Supergroup, Western India. Int. Geol. Rev..

[27] Oehlert AM, Swart PK (2014). Interpreting carbonate and organic carbon isotope covariance in the sedimentary record. Nat. Commun..

[28] Liu LH, Ma YS, Liu B, Wang CL (2017). Hydrothermal dissolution of Ordovician carbonates rocks and its dissolution mechanism in Tarim Basin, China. Carbonates Evaporites.

[29] Price RE (2013). Processes influencing extreme As enrichment in shallow-sea hydrothermal fluids of Milos Island, Greece. Chem. Geol..

[30] Zhu D, Meng Q, Jin Z, Liu Q, Hu W (2015). Formation mechanism of deep Cambrian dolomite reservoirs in the Tarim basin, northwestern China. Mar. Pet. Geol..

[31] Nowrouzi Z, Moussavi-Harami R, Mahboubi A, Mahmudy Gharaie MH, Ghaemi F (2014). Petrography and geochemistry of Silurian Niur sandstones, Derenjal Mountains, East Central Iran: Implications for tectonic setting, provenance and weathering. Arab. J. Geosci..

[32] Wei X, Wang S, Ji H, Shi Z (2018). Strontium isotopes reveal weathering processes in lateritic covers in southern China with implications for paleogeographic reconstructions. PLoS ONE.

[33] Cloutier V, Lefebvre R, Therrien R, Savard MM (2008). Multivariate statistical analysis of geochemical data as indicative of the hydrogeochemical evolution of groundwater in a sedimentary rock aquifer system. J. Hydrol..

[34] Zhang H, Xu G, Chen X, Mabaire A (2019). Hydrogeochemical evolution of multilayer aquifers in a massive coalfield. Environ. Earth Sci..

[35] Zhang H (2020). Groundwater hydrogeochemical processes and the connectivity of multilayer aquifers in a coal mine with karst collapse columns. Mine Water Environ..

[36] Zhang H (2020). Identification of hydrogeochemical processes and transport paths of a multi-aquifer system in closed mining regions. J. Hydrol..

[37] Meng X, Wan Y, Bai X (2018). Characterization and origin of a dolomite reservoir in weathering crust: Example from Ordovician in the Tabamiao Region, Northern Ordos, China. Carbonates Evaporites.

[38] Xiao D (2019). Discovery of syngenetic and eogenetic karsts in the Middle Ordovician gypsum-bearing dolomites of the eastern Ordos Basin (central China) and their heterogeneous impact on reservoir quality. Mar. Pet. Geol..

[39] Carpenter SJ, Lohmann KC (1995). δ18O and δ13C values of modern brachiopod shells. Geochim. Cosmochim. Acta.

[40] Hendy CH, Wilson AT (1968). Palaeoclimatic data from speleothems. Nature.

[41] Nelson CS, Smith AM (1996). Stable oxygen and carbon isotope compositional fields for skeletal and diagenetic components in New Zealand Cenozoic nontropical carbonate sediments and limestones: A synthesis and review. NZ J. Geol. Geophys..

[42] Yang F, Bao Z, Zhang D, Jia X, Xiao J (2017). Carbonate secondary porosity development in a polyphase paleokarst from Precambrian system: Upper Sinian examples, North Tarim basin, northwest China. Carbonates Evaporites.

[43] Gasparrini M, Bechstädt T, Boni M (2006). Massive hydrothermal dolomites in the southwestern Cantabrian Zone (Spain) and their relation to the Late Variscan evolution. Mar. Pet. Geol..

[44] Huang S (2008). Evolution of strontium isotopic composition of seawater from Late Permian to Early Triassic based on study of marine carbonates, Zhongliang Mountain, Chongqing, China. Sci. China Ser. D Earth Sci..

[45] Arthur MA, Dean WE, Pratt LM (1988). Geochemical and climatic effects of increased marine organic carbon burial at the Cenomanian/Turonian boundary. Nature.

[46] Kuypers MMM, Pancost RD, Damsté JSS (1999). A large and abrupt fall in atmospheric CO_2_ concentration during Cretaceous times. Nature.

[47] Scholle, P. A. & Arthur, M. A. *Carbon Isotope Fluctuations in Cretaceous Pelagic Limestones: Potential Stratigraphic and Petroleum Exploration Tool1*. Vol. 64 (1980).

[48] Keith ML, Weber JN (1964). Carbon and oxygen isotopic composition of selected limestones and fossils. Geochim. Cosmochim. Acta.

[49] Seward D (1978). Palaeosalinities and palaeotemperatures from carbon and oxygen isotopes of carbonate shells in three quaternary formations, Wanganui Basin, New Zealand. Palaeogeogr. Palaeoclimatol. Palaeoecol..

[50] Urey HC (1948). Oxygen isotopes in nature and in the laboratory. Science.

[51] Epstein S, Mayeda T (1953). Variation of O18 content of waters from natural sources. Geochim. Cosmochim. Acta.

[52] Zhang H (2019). Hydrogeochemical characteristics and groundwater inrush source identification for a multi-aquifer system in a coal mine. Acta Geol. Sin. Engl. Ed..

[53] Gao, D., Lin, C., Hu, M., Yang, H. & Huang, L. Early Mesozoic intracontinental deformation in the eastern North China Block: Implication for an indentation model of North China to South China blocks. **52**, 8–21 (2017).10.1002/gj.3058

[54] Wei X (2018). Paleogeomorphy evolution of the Ordovician weathering crust and its implication for reservoir development, eastern Ordos Basin. Petrol. Res..

[55] Jia Z-Z, Lin C-Y, Ren L-H, Dong C-M (2016). Selective dissolution of eodiagenesis cements and its impact on the quality evolution of reservoirs in the Xing’anling Group, Suderte Oil Field, Hailar Basin, China. Petrol. Sci..

[56] Xiao D (2016). An inland facies-controlled eogenetic karst of the carbonate reservoir in the Middle Permian Maokou Formation, southern Sichuan Basin, SW China. Mar. Petrol. Geol..

[57] Frost EL, Kerans C (2010). Controls on syndepositional fracture patterns, Devonian reef complexes, Canning Basin, Western Australia. J. Struct. Geol..

[58] La Bruna V (2020). Structural diagenesis of shallow platform carbonates: Role of early embrittlement on fracture setting and distribution, case study of Monte Alpi (Southern Apennines, Italy). J. Struct. Geol..

[59] Lavenu APC, Lamarche J, Gallois A, Gauthier BDM (2013). Tectonic versus diagenetic origin of fractures in a naturally fractured carbonate reservoir analog (Nerthe anticline, southeastern France). AAPG Bull..

[60] Fu H (2017). Forming mechanism of the Ordovician karst carbonate reservoirs on the northern slope of central Tarim Basin. Nat. Gas Ind. B.

[61] Todaro S, Hollis C, Di Stefano P (2016). Spongy-like porosity in peritidal carbonates: An interaction of cyclic sea-level oscillations, fresh water supply and sediment texture. Sed. Geol..

[62] Baceta JI, Wright VP, Beavington-Penney SJ, Pujalte V (2007). Palaeohydrogeological control of palaeokarst macro-porosity genesis during a major sea-level lowstand: Danian of the Urbasa-Andia plateau, Navarra, North Spain. Sedim. Geol..

[63] Li H, Cai C (2017). Origin and evolution of formation water from the Ordovician carbonate reservoir in the Tazhong area, Tarim Basin, NW China. J. Petrol. Sci. Eng..

[64] Mazzullo SJ, Harris PM (1992). Mesogenetic dissolution: its role in porosity development in carbonate reservoirs 1. AAPG Bull..

[65] Yuan W, Yang Z (2015). The Alashan Terrane did not amalgamate with North China block by the Late Permian: Evidence from Carboniferous and Permian paleomagnetic results. J. Asian Earth Sci..

[66] Hao F (2015). The fate of CO2 derived from thermochemical sulfate reduction (TSR) and effect of TSR on carbonate porosity and permeability, Sichuan Basin, China. Earth Sci. Rev..

[67] Wu M, Wang Y, Zheng M, Zhang W, Liu C (2007). The hydrothermal karstification and its effect on Ordovician carbonate reservoir in Tazhong uplift of Tarim Basin, Northwest China. Sci. China, Ser. D Earth Sci..

[68] Wang Z, Zhang Y, Tao X, Zhu B, Luo C (2015). Genesis of the Ordovician fluorite and its geological significance in central uplift of the Tarim basin, China. Mineral. Petrol..

[69] Li J, Wang Y, Liu C, Dong D, Gao Z (2016). Hydrothermal fluid activity and the quantitative evaluation of its impact on carbonate reservoirs: A case study of the Lower Paleozoic in the west of Dongying sag, Bohai Bay Basin. Petrol. Explor. Dev..

